# Dopamine genes are linked to Extraversion and Neuroticism personality traits, but only in demanding climates

**DOI:** 10.1038/s41598-017-18784-y

**Published:** 2018-01-29

**Authors:** Ronald Fischer, Anna Lee, Machteld N. Verzijden

**Affiliations:** 1 School of Psychology, Victoria University of Wellington, Wellington, New Zealand; 2 Department of Molecular Biology and Genetics - DANDRITE, Aarhus University, Aarhus, Denmark

## Abstract

Cross-national differences in personality have long been recognized in the behavioural sciences. However, the origins of such differences are debated. Building on reinforcement sensitivity theories and gene-by-environment interactions, we predict that personality trait phenotypes linked to dopaminergic brain functions (centrally involved in reward processing) diverge most strongly in climatically stressful environments, due to shifts in perceived rewards vs risks. Individuals from populations with a highly efficient dopamine system are biased towards behavioural approach traits (Extraversion and Emotional Stability) due to higher perceived reward values, whereas individuals from populations with a less efficient dopaminergic system are biased towards risk avoidance. In temperate climates, we predict smaller phenotypic differences due to overall weakened reward and risk ratios. We calculated a population-level index of dopamine functioning using 9 commonly investigated genetic polymorphisms encoding dopamine transporters and receptors, derived from a meta-analysis with data from 805 independent samples involving 127,685 participants across 73 societies or territories. We found strong support for the dopamine gene by climatic stress interaction: Population genetic differences in dopamine predicted personality traits at the population level in demanding climates, but not in temperate, less demanding climates, even when controlling for known correlates of personality including wealth and parasite stress.

## Introduction

Persons vary in how they behave when faced with social or economic choices, which can have far reaching consequences for their life’s outcomes. Human personality traits, in particular those regulated by behavioural approach vs avoidance motivation such as Extraversion and Neuroticism^1–4^, vary both within human populations, and between human populations across the globe^5–9^. Attempts to explain such differences range from genetic to cultural hypotheses, and there is evidence that both genetic and cultural variation affect variation in personality^8,10–12^. Personality traits are heritable^10^, and genetic variation in the production and uptake of neuromodulators such as dopamine may play some role^2,4,13^. Dopamine activity has been experimentally linked to differences in personality traits through a variety of methods^14^, yet, the relationships between genes regulating dopamine activity and global personality phenotypes has been less than consistent^15^. This may be because a large vector of environmental factors (e.g., parental support, negative life events, resource availability) also affect the development of personality traits, resulting in different phenotypes despite similar genotypes depending on the environmental circumstances. This is commonly termed phenotypic plasticity^16^. Taken one step further, this implies that different genotypes may respond differently to environmental factors, resulting in a pattern of genotype by environment interactions^17,18^. Not just individual environmental differences may be seen to affect the development of personalities, contextual factors that vary at the population level scale such as climatic demands can contribute to how individual personalities develop, which in turn may affect population level patterns of personality traits. Indeed, climatic demands have been shown to affect a variety of psychological functions and behavioural traits in modern human populations^19^, which are influencing personality traits on a broader geographical scale. Importantly, these broader climatic effects can also differentially impact the personality development of various genotypes, causing a greater phenotypic variation between genotypes in some climates than others. Here we investigate the existence of such interaction between genetic predisposition (dopaminergic system genes) for personality phenotypic traits and the effects of climatic demands on the development of the phenotype, predicting that there is greater variability between genotypes in more demanding climates compared to benign climates. In other words, we investigate a genotype by environment interaction (GxE) where the greatest effect of the environment is expected in more demanding climates, causing greater divergence in the developmental outcomes of the different genotypes. We link these variables to differences in two key personality traits: Extraversion and Neuroticism/Emotional Instability^1–3^.

## Personality Structure

Across human and animal studies, there is a general recognition that personality is structured into a number of basic dimensions, with traits differentiating broad behavioural approach vs avoidance motivation emerging in practically all personality models in both human and animal studies^20,21^. In humans, these two motivational systems correspond to extraversion (vs introversion) and neuroticism (with emotional stability as the opposing pole)^2,4^. Extraversion is characterized by a general motivation to approach new situations, high levels of energy and sociability as well as positive emotionality, making it the prototypical behavioural approach motivational trait. Individuals high in neuroticism react more strongly to stressful stimuli, are more likely to avoid novel situations and potentially aversive stimuli. It is therefore a trait strongly regulated by avoidance motivation systems. Extraversion and neuroticism are distinct traits that systematically vary within and between populations^6,22^. For Extraversion, across population variation typically accounts for between 3 and 7% of the total variation and about 3% and 10% of the variance in Neuroticism^22,23^. Asian samples have lower Extraversion than European and North American samples, whereas East Asian, Southern European and South American populations show higher Neuroticism means^5,22,23^.

There is evidence that variation in genes regulating a dopaminergic brain system modulate processing differences in the striatum, prefrontal cortex areas and limbic system, which are centrally involved in decision-making, reinforcement learning and risk assessment^20,21,24,25^. Dopamine is one of the main neurotransmitters within the behavioural approach system^1,2^, with multiple correlational and experimental methods showing the relevance of dopamine for extraversion and neuroticism-type behavioural traits^13,14^. Furthermore, variants of dopamine genes have been associated with variation in personality traits in both clinical and general populations, although the direction and stability of these associations is less clear^14,15,26^. This clearly points to a need to pay greater attention to environmental conditions that may influence gene expression^27^, since dopamine is clearly involved in regulating brain systems that control cognitive and emotional decision processes that underlie both Extraversion and Neuroticism^14,25^.

Recent studies have shown that these functionally important genes vary not only between individuals but the frequency of their occurrence also differs between human populations^28^. This raises the possibility that the same gene complexes may also contribute to variation in previously observed population-level differences in personality traits, in particular when interacting with the larger environmental context. Carriers of the same genetic variants may show phenotypic plasticity, which can result in highly different developmental outcomes depending on the environmental circumstances. Recently accumulated evidence from various labs strongly suggests that carriers of variants of the polymorphic gene for a dopamine receptor (DRD4) develop different personality traits, depending on the social circumstances during childhood^18^. Indeed, the lack of direct gene effects noted in previous meta-analyses might be due to these environmental contingencies^27^.

We argue that such interactions also act at a wider scale: ecological conditions and genetic predispositions interdependently influence phenotypic personality traits in a classic gene-environment interaction pattern. We focus on climate as one core feature of the local environment which influences a swath of processes related to body function and survival. We control for wealth, parasite stress levels as well as climate-economic conditions as alternative explanations of cross-cultural differences in personality trait levels.

## Climatic Stress

The role of climatic stress has been recognized as a major contributor to the expression of social and psychological phenomena in humans^19^ and other animals^29^. We define climatic demand as the deviation from an average monthly temperature of 22 °C, as a relative optimum for human functioning^19^. Climatic demands directly and negatively impact on core needs of humans, including the need to maintain a core body temperature of approximately 37 °C. Secondarily, climatic demands impose restrictions in access to food sources, which negatively impacts the ability to have regular access to high caloric food sources to sustain body functions^30,31^. In addition, in climates with higher climatic demands (particularly in hot climates), greater parasite stresses prevail that negatively impact on body functions^32^. In more demanding climates (either hot or cold), different strategies might be effective to get access to resources (clothing, shelter, heating or cooling systems, etc.) and ward off other threats to stay healthy. Stress, from temperature or parasites, is known to affect physiology, and there is a strong link between physiology, such as metabolic rate, and behavioural activity.

## Dopaminergic reward processes leading to phenotypic plasticity

Both behavioural approach and avoidance strategies might be adaptive in high climate stress environments, depending on the reward parameters of the biological system. Approach behaviour is likely to yield additional resources through the exploration of novel situations, yet it also increases risk due to conflict and violence, predators, diseases and injuries^23,33^. On the other hand, while avoidance behaviour reduces the overall risk, it also decreases the chances of obtaining additional resources that might increase fitness. Dopaminergic processes are centrally involved in reward processing, and genetic variation in dopamine production or sensitivity may mediate the personality traits a person develops^3^, by reinforcement (level of reward sensations) through positive or negative experiences. A highly functional dopaminergic system is hypothesized to be associated with Extraversion and Emotional stability, while a a less functional dopaminergic system where dopamine transmission or re-uptake is slower is more associated with Introversion and Neuroticism^4^. In benign environments most suitable for human thriving, resources are more abundant (decreasing the reward incentive for approach motivated individuals) and risks are less severe (decreasing the risk component for avoidance motivated individuals), therefore decreasing the motivational differences between the two groups. Thus, positive and negative experiences might be processed differently by a person with a highly functional dopaminergic system when they are stressed or not, leading to different personality profiles under stressful compared to benign circumstances. We propose that in highly demanding climates individuals with a highly functional dopaminergic system are more strongly positively reinforced by rewards than the negative experience of risk. Thus, they will develop behavioural tendencies towards stronger approach motivation. In contrast, in high stress environments, individuals with a less down-regulated dopaminergic system might shift more towards avoidance behaviours, because of a higher perceived risk in a climatically demanding environment.

In other words, following conventions of phenotypic plasticity research, we expect greater differentiation of phenotypes in high stress environments, due to the differential reward vs risk sensitivity associated with the phenotypes. In climates optimal for human functioning, the relative contrast of rewards and risks is less drastic and therefore, phenotypic differences in traits should be weakened. Thus, genetic differences are more amplified in the developmental outcome at the climatic demanding end, leading to greater phenotypic variation in personality traits.

## Dopaminergic System Genes

**Figure 1:**
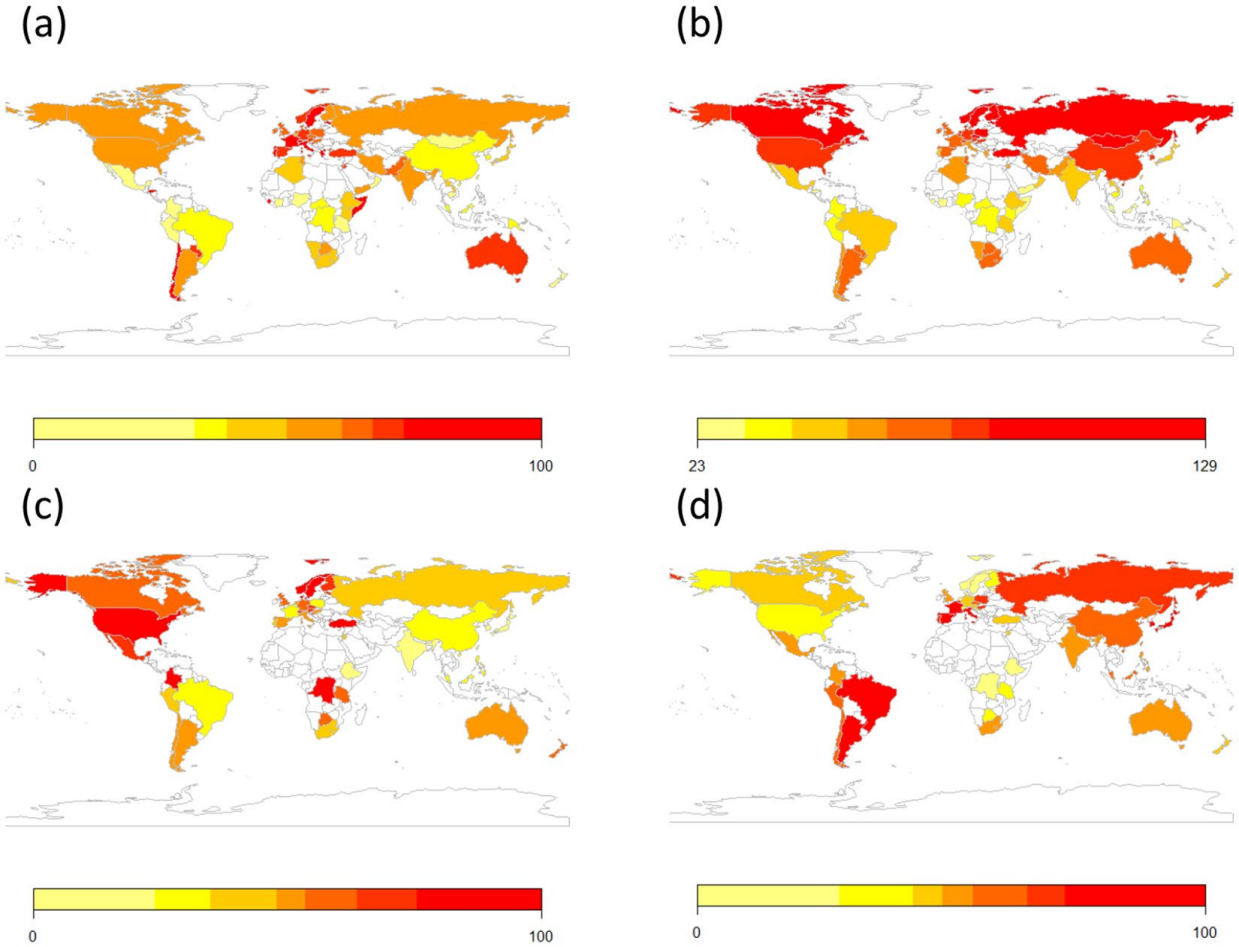
Panel (a) Dopamine system index for all nations for which data was available (see Supplementary Table 1 for data). The index was normalized and standardized to range between 0 (lowest dopamine functioning) and 100 (highest observed dopamine functioning). Panel (b) Climatic demands for all nations included in the analysis. Climatic stress was computed as the sum of the deviations from 22 °C (ca. 72F) for the lowest and highest temperatures in the coldest month, and the lowest and highest temperatures in the hottest month. Panel (c) Joint distribution of the combined Extraversion personality scores for all nations where Dopamine data was available. The rescaled data (varying between 0 and 100) from the three personality data sources was linearly combined. Panel (d) Joint distribution of the combined Neuroticism personality scores for all nations where Dopamine data was available. The rescaled data (varying between 0 and 100) from the three personality data sources was linearly combined. The data was plotted using the rworldmap package in R.

We constructed an overall index of dopamine functioning from published research, overcoming previous limitations with single gene loci^34^. Previous research has focused on either the role of a specific candidate gene or used genome-wide association studies. Yet, personality is a complex phenotypic trait and many genes individually may contribute small amounts of variance to the overall phenotype^12,34^. Therefore, we reasoned that analysing a combination of functionally relevant genes might be a more fruitful method for understanding gene-phenotype effects in the personality domain, using a so-called gene systems approach^34^. We focused on polymorphisms associated with the dopamine system that a) have been treated as candidate genes influencing personality traits in previous research (e.g., influencing the efficiency and density of the dopamine transporter and receptors in the brain, thereby regulating the overall functioning of the dopaminergic pathways), and which b) have been studied in diverse population samples, with data from at least 10 different ethnic or cultural samples being available. We were constrained by the availability of primary studies, but we aimed to include transporter, receptor and promotor genes to create an index capturing the overall functionality of the dopaminergic brain system. For this reason, we examined single nucleotide polymorphisms (SNP) and did not include variable number tandem repeats (VNTRs) such as the commonly studied DRD4 48 bp repeat polymorphism. We did include the DAT1 VNTR^35^ because there are fewer functional variants and the major functional distinction is between the 9 and 10 base pair repeat DRD1 and other variants are rare. The method section describes the covariation of the individual SNPs (and one VNTR) that allowed us to create a single index. Our index includes data from 805 independent samples involving 127,685 participants across 73 societies or national territories (listed in Table 1 of the Supplementary data). The overall index provides the currently most comprehensive overview on population-genetic differences in dopamine genes around the globe (see Fig. 1, panel a for the global distribution). Using such a large sample allows us to overcome problems with the previously noted small sample problem in population-genetic analyses.

To test our hypotheses, we used personality scores from three independent data-bases which included data from students, employees and general population samples, which allows us to overcome challenges in terms of replicability and validity. Having independent sources measured with different instruments, across independent samples, and time horizons enhances the confidence in the reported patterns.

## Results

**Figure 2:**
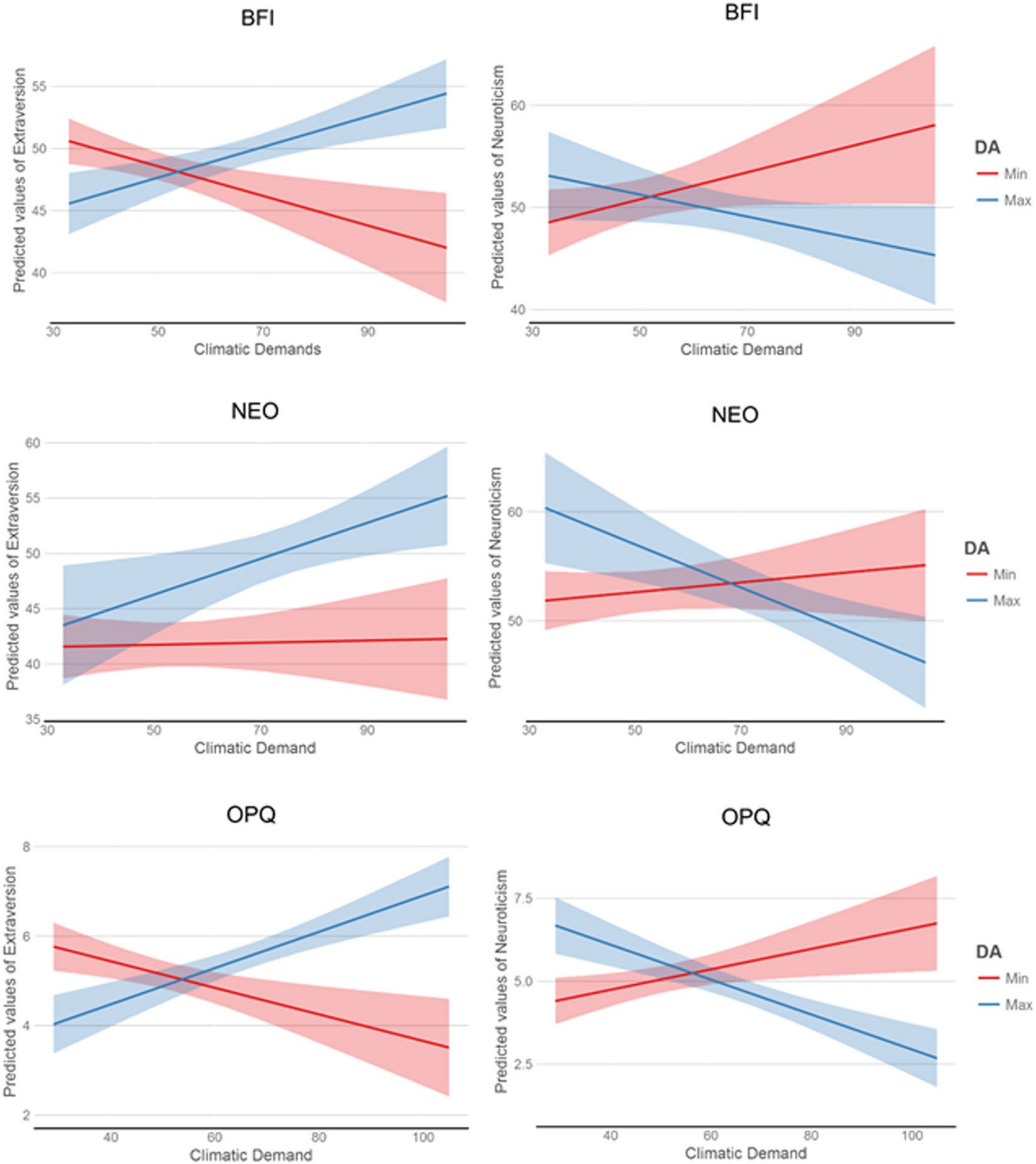
Left Panel: The interactive effects of the dopamine index (DA) and climatic demands on Extraversion. There is greater phenotypic plasticity in high climatic stress environments. Higher dopamine index scores are associated with more Extraversion in climatic stressful environments, but no relationship is found for low dopamine index scores when measuring personality with (**a**) the BFI, (**b**) the NEO-PI-R and (**c**) the OPQ. Right Panel: The interactive effects of the dopamine index and climatic stress on Neuroticism. There is greater phenotypic plasticity in high climatic stress environments. Higher dopamine index scores are associated with lower Neuroticism when measured with (**a**) BFI, (**b**) NEO-PI-R, and (**c**) OPQ in environments with high climatic stress, compared to benign climatic environments.

The proposed interaction between climatic stress and the dopamine genetic index was significant in five out of the six models (see Table 1). Higher scores on the dopamine index were associated with higher levels of Extraversion and lower levels of Neuroticism, but only in climatically demanding environments. Figure 2 displays the simple slope effects at 1 SD above and below the mean, showing that differences in personality between dopamine efficient and less efficient was highest in conditions of climatic stress, consistent with phenotypic plasticity. Using bootstrap sampling with 1,000 random replacement samples, the interaction effect remained significant in five of the six analyses (*P* < 0.05). We conducted a multivariate outlier test and found no statistically significant outlier in our dataset (P > 0.05).

**Table 1:** Results of linear model analysis predicting Neuroticism and Extraversion nation level scores from climatic stress and the population dopamine index.

	N			E		
	NEO-PI-R	OPQ	BFI	NEO-PI-R	OPQ	BFI
Intercept	53.87	4.82	51.11	44.50	5.09	48.17
Dopamine	0.56	−0.10	−0.58	2.00*	0.13	0.47
Climatic Stress	−1.37	0.34**	0.18	1.75*	0.20*	0.15
Dopamine × Climatic stress	−1.81*	−0.50**	−1.51*	1.14	0.42**	1.53**
ΔF interaction	5.15*	11.02**	2.92*	1.80	13.35**	9.19**
F	3.14	7.83	1.12	9.80	7.81	3.96
df	3, 23	3, 28	3,34	3,23	3, 28	3,34
Explained variance (R²*100)						
Main effects	13.14	24.20	1.14	52.67	19.62	5.83
Interaction	15.90	21.41	7.83	3.44	25.94	20.04
Full model	29.04	45.61	8.97	56.11	45.56	25.87

One major caveat is that nations are not independent and that there might be population structures underlying the genetic data. Therefore, we added continent as a dummy-coded predictor variable. None of our main results were affected by adding continent dummy codes. This overall strongly supports our hypothesis. Probing the interaction further, simple slope tests revealed that only the regression slope for the higher score on the dopamine index was significant (five of the six slopes were significant at *P* < 0.05). This suggests that a highly functional dopaminergic system at the population level are associated with more behavioural approach traits with increasing climatic stress, but that climatic demands do not affect phenotypes in populations with a less efficient dopaminergic system.

Next, we tested whether including wealth and parasite stress would render the interaction non-significant. Both wealth and parasite stress have been shown to affect personality differences across nations, with greater wealth and lower parasite stress being associated with more extrovert and emotionally stable personality traits^22,23,32^. Therefore, controlling for these effects, we test whether our proposed interaction adds any additional explained variance in the statistical prediction of personality scores. The genotype by environment interaction remained significant for four of the six models (*P* < 0.05, plus a statistical trend for Extraversion measured with the BFI: *P* = 0.07). On average, the interaction explained an additional 12.04% of the variance in personality trait scores at the population level over and above wealth and parasite stress (ranging from 3% to 23% for individual instruments). The current model captures additional variance that has not been covered by previous analyses of personality at the societal level.

A second competitive test is against climato-economic theory of culture^19,36^, which predicts that climatic demands provide challenges to humans, which need to be met by adequate resources (e.g., wealth) to allow personal growth. In the absence of adequate resources, behavioural reactions are singularly focused on mitigating threats and undermine personal growth. In line with this reasoning, a number of studies have shown support for these predictions^9^. Controlling for the interaction between climatic demands and wealth before testing our predicted interactions between climatic demands and dopamine therefore provides a strong empirical challenge. After controlling for the climate-economic interaction, four of the six predicted interactions were still statistically significant and one interaction (for Neuroticism when measured with the BFI) revealed a trend (*P* = 0.14). Our predicted interaction contributed on average an additional 14.85% and 5.33% of the variance in Extraversion and Neuroticism, respectively.

To test whether the interaction was specifically targeted towards Extraversion and Neuroticism, we ran additional analyses examining the interaction for the other personality traits measured by the same instruments (Conscientiousness, Agreeableness, Openness/Intellect). After adjustment for multiple significance tests, one effect was statistically significant (average *P*-value was 0.61): the interaction between dopamine and climatic demands was significant for Openness/Intellect when measured with the OPQ (*B* = 0.34, *P* < 0.005). The interaction replicated the interaction found for Extraversion. Extraversion and Openness/Intellect are conceptually related because Extraversion taps into behavioural flexibility, whereas Openness/Intellect taps into cognitive flexibility^37,38^. It is therefore compatible with our argument, even though we did not predict this interaction (since Openness/Intellect is a more cognitive personality domain).

## Discussion

In summary, we provide first evidence that population-genetic differences in the dopamine-system are related to personality trait differences in Neuroticism and Extraversion, but only in contexts where there is high climatic stress. Our data shows that genotypes with a higher score in the dopaminergic brain function index confers higher Extraversion and lower Neuroticism in climatically demanding climates, but not in regions that are classified as imposing a lower climatic stress. This pattern was highly consistent between three different instruments used in measuring personality across the world. This linear analysis suggested that populations with the same genetic predispositions develop different phenotypes depending on environmental gradients under climatic demand. In contrast, dopaminergic systems with slower dopamine uptake, in high climatic stress environments, were not associated with different phenotypes compared to benign climatic environments. This suggests either weakened reward processing in individuals with a lower score in the dopaminergic brain function index or some other mechanisms may mitigate these climatic stressors in these populations.

That both genes affecting dopaminergic brain pathways and climatic demand interact with each other in how they affect personality traits is theoretically important because it offers both a plausible mechanism underlying cultural differences in personality traits and points to a possible reason for the often noted absence of correlations between personality and specific genes^39^. We propose that population genetic differences play a role in explaining population-wide differences in personality traits, but that the relative importance of genetics depends on environmental variables, including climatic demands due to their broad impact on various biological functions. As discussed above, we focus on climatic demands because it affects a wide range of biological processes related to homeostatic regulation, food security, risks of injury, and prevalence of disease stress^19^ that have significant implications for approach versus avoidance behaviours, leading to differential expression of trait phenotypes.

Importantly, our population genetic approach, focusing on interactions with environmental variables, also points to one possible reason why previous candidate gene approaches were less likely to produce reproducible results across different populations. The overall stress levels in an environment might provide a background context, which influences how genes are expressed phenotypically. Studies on personality traits that focus on gene-environment interactions typically focus on stressful life events or other proximal variables that affect individuals^27^. Yet, the general stress level prevalent in an environment, which affects to various degrees all individuals within a given population, may exert some influence that has remained unexamined. These effects might be exacerbated if these stressors are not met by sufficient material resources^19,23^. This is an important avenue for studying gene-personality relations in the future.

At the same time, the presence of gene-environment interactions is one likely candidate to explain why there have been a good number of null-results in the literature on gene-personality linkages. The phenomenon that (temperature) stress may expose higher rates of phenotypic variation has been noted in the field of evolutionary biology. In the evolutionary model species *Drosophila melanogaster* fruit flies, various morphological and physiological traits showed higher heritability and greater variance under less benign circumstances^40^, yet the processes outside the laboratory might be more complex and the effects of stress may not universally increase phenotypic variation^41,42^.

We used a genetic systems approach to test for interactions between population genetic differences and the thermal environment. Current approaches emphasize candidate gene approaches or genome-wide association studies, which have specific limitations^27,34^. Personality is a complex trait and plausibly many different genes with small effect contribute to the phenotype. Genetic systems approaches may thus provide new insights. We provide one such index focusing on SNPs (and one VTNR) in the dopamine system. With the increasing availability of genetic information at the population level, systems approaches can be refined and extended. Our current index (see Fig. 1, Methods) can be used for exploring other phenotypic associations at the population level in future studies. With increasing genetic data available, improvements in such indices separating homozygote and heterozygote frequencies would also be feasible, adding to the power of this approach. The data presented here focused on dopamine because it is an important neurotransmitter involved in personality traits and sufficient data on multiple genes regulating dopamine were available across multiple sites. However, human personality is almost certainly affected by other functional pathways. Thus, future work on candidate genes modulating other neurotransmitters of importance to personality, such as serotonin^3,4,17^ is likely a fruitful avenue of investigation.

## Method

### Sample

We conducted an online literature search using PSYCINFO and PUBMED published till December 2012, using the following search terms “dopamine polymorphism”, “dopamine receptor polymorphism”, “dopamine transporter polymorphism”, “dopamine gene variant”, “dopamine receptor genotype”, “dopamine transporter genotype”, “DAT1 genotype”, “DRD1 genotype”, “DRD2 genotype”, “DRD3 genotype”, “DRD4 genotype”, and “DRD5 genotype”. This search returned 9,058 articles in total. We focused on regionally or ethnically identifiable human populations with no current clinical health problems. For this reason, we excluded a) non-human animal studies, b) patient or clinical samples without healthy controls (control groups with no clinical symptoms were included), c) pedigree studies (due to unclear status of the wider representativeness of the specific samples in the larger population), d) mixed samples with unclear ethnic or national status and e) articles published in other languages than English. Meta-analyses and reviews were scanned for possibly relevant studies. If an article did not provide sufficient information to code relevant SNP frequencies, it was also discarded. All studies were checked manually to ensure that data from the same study population reported across multiple articles was only included once. If this was the case, we selected the article with the highest N or the most detailed reporting of allele type frequencies or genotypes to allow coding for our purposes. A total of 936 articles were identified in this step. To identify unpublished papers, we searched the ALFRED database^43^. We used search terms related to all relevant dopamine genes: dopamine receptors one to five (“DRD1”, “DRD2”, “DRD3”, “DRD4”, “DRD5”), and the dopamine transporter (“DAT1”). This search resulted in an extra 32 studies being added to the database.

We did include DRD4 as a search term because there are multiple polymorphisms that have been discussed. The most commonly discussed polymorphism (the 48 bp repeat) has various different repeats that have been shown to have functionality for personality, some of which have only recently been reported^44^. Many older studies did not provide sufficient information to code functionally relevant data. The complexity of having multiple functionally relevant variants that differ widely across populations together with the limited information available did not allow us to include it in our dopaminergic brain function index. We did include the DAT1 VNTR^35^ because there are fewer functional variants and the major functional distinction is between the 9 and 10 base pair repeat. Therefore, we coded the frequency of at least one 9 repeat allele as indicating higher dopaminergic functioning^45^.

After initial coding of all articles and reports, we excluded polymorphisms that had been only reported in 10 or fewer populations. The final database consisted of 551 articles or unpublished reports, reporting data from 805 independent samples involving 127,685 participants. For case and clinical studies, only data from healthy controls was used. An overview of the samples and locations is provided in Table 2.

**Table 2:** Overview of the selected dopamine genes, specific SNP, samples sizes and available samples.

Gene	Polymorphism	Samples	Total N	Nations
DAT1	rs28363170 (‘3 40 bp VNTR)	265	43,502	46
DRD2	rs1800497 (Taq1A)	388	65,617	59
DRD2	rs1079597 (Taq1B)	199	15,785	55
DRD2	rs1800498 (Taq1D)	120	10,814	37
DRD2	rs6275 (C939T)	122	10,404	46
DRD2	rs6277 (C957T)	114	20,375	46
DRD2	rs1799732 (141 C in/del)	91	19,074	35
DRD3	rs6280 (ser9gly)	237	28,796	63
DRD4	rs1800955 (C521T)	72	13,795	32

*Note*: Total N and number of studies varies due to multiple samples including more than one SNP.

The personality data restricts the geographic resolution of our analysis, since personality data was only available at the country level. If genetic data from multiple ethnic or cultural groups were available from a specific country, we averaged and weighted each sample by sample size. Therefore, the genetic data presents the average of the allele frequencies across the various samples. In the case of the US and the UK, we only used Caucasian and White British samples. Some diaspora populations that had recently migrated were coded in terms of country of origin. Additional analyses were conducted at the ethnic group level nested within countries. The overall results were replicated. For ease of presentation we report the coarser nation-level data. We coded the allele frequency and then aggregated the data to the nation level weighted by sample size. More information on each study and sample are available in the supplementary files.

### Dopaminergic Brain System Index

We first examined the relative co-occurrence of the various polymorphisms at the sample level and at the population level. A principal component analysis of the smoothed correlation matrix at the genetic sample level yielded three principal components that explained a total of 88% of the variance. The first component explained 47% and had an Eigenvalue of 4.25. At the population level, a similar structure emerged (Tucker’s *φ* = 1.00, 0.99 and 0.99 for the first, second and third components) explaining 85% of the variance. The first component at the population level explained 47% of the variance (Eigenvalue = 4.26). The loadings on the first principal component across study and population level were basically identical (r = 0.99). Cronbach’s alpha at the population level demonstrated high reliability (*α* = 0.85, average *r* = 0.39).

The loadings on the single component are in line with interpretations of a higher score in terms of dopaminergic functioning at the neural level. The highest loading polymorphism is DRD2 Taq1A (A1 allele, *λ* = −0.89), followed by DRD2 rs6277 (C allele, *λ* = −0.87), DRD2 Taq1D (D1 allele, *λ* = 0.86), DAT1 VNTR (9-repeat allele, *λ* = 0.75), DRD2 Taq1B (B1 allele, *λ* = −0.67), DRD3 ser9gly (ser allele, *λ* = 0.50), DRD4 C521T (T allele, *λ* = 0.63), DRD2 141C-in/del (insert, *λ* = 0.44) and DRD2 rs6275 (C allele, *λ* = 0.34). These alleles have been shown to be involved in a range of behavioural and emotional phenotypes^26,34,45–50^, even while the biochemical mechanisms are still underexplored. Our results indicate that these polymorphisms systematically covary across both samples and populations, showing one strong first factor capturing overall differences in dopaminergic brain functioning. Data for all nations is listed in Supplementary Table 1.

### Climatic stress

Climatic demands were operationalized as the sum of the deviations from 22 °C (ca. 72F) for the lowest and highest temperatures in the coldest month, and the lowest and highest temperatures in the hottest month^19^. This index has been based on extensive biological and physiological research that suggests this to be an optimal temperature for human functioning and flourishing. One potential issue with using this index is temperature variation in nations covering multiple latitudes. Van de Vliert^19^ demonstrated that using these unadjusted effects provides more conservative estimates of climate effects. We follow this approach and did not adjust for latitude differences within nations.

### Personality

We used three different data-bases, which have shown adequate reliability, validity and cross-cultural measurement invariance. First, the NEO-PI-R was developed by Costa and McCrae^51^. It is the most widely used instrument to measure the Big Five. The instrument includes 240 items, containing 30 8-item facet scales, capturing Neuroticism (N), Extraversion (E), Openness to Experience (O), Agreeableness (A), and Conscientiousness (C). Answers are scored on a five-point Likert scale; from strongly disagree to strongly agree. The instrument has been translated and validated in over 30 languages. We used N and E national means from self-reports in student and general population samples^52,53^. Matching data with our dopamine index was available from 27 nations. Second, the Big Five Inventory (BFI) was developed by Benet-Martinez and John^54^ as a 44-item short assessment of the same five personality dimensions. Self-responses are scored on a similar 1 (disagree strongly) to 5 (strongly agree) Likert scale. We used data collected and validated by Schmitt *et al*.^22^ from college students in 56 nations. We had overlapping data with our dopamine index for 38 nations. Finally, we used data measured using the OPQ32i^55^. The OPQ32i consists of 104 sets of item quads (four statements are presented that belong to different personality scales) forming 32 different scales. For each quad, the respondent chooses one statement as best representing him or her, and one statement as least representing his or her. These faking resistant scores were reconverted to normative scores and the 32 specific scales have been mapped onto the Big Five personality taxonomy. The data we use is based on job applications or personality assessments conducted in organizational settings, therefore, it is data that is commercially used and has been shown to be of adequate cross-cultural validity to be used for organizational decision-making^56^. Data from 32 countries was available.

### Control variables

We used averaged normalized estimates of Gross Domestic Product per capita (expressed in product purchase parity) from 1985 to 2005^57^. This average was used to better capture the long-lasting effects of economic development on personality factors. Wealth has been shown to correlate with mean level personality traits^5,58^, with typically higher means of Extraversion and lower means of Neuroticism in wealthier nations. In our total sample for which we had personality data, wealth correlated (*r* = 0.42, *P* < 0.01) with climatic stress and (*r* = 0.36, *P* < 0.05) with the dopamine index.

We also included parasite stress as a control variable because it has been shown to correlate with mean personality traits^23,32^. We used historical pathogen prevalence rates^59^ including rates for leishmanias, schistosomes, trypanosomes, leprosy, malaria, typhus, filariae, dengue and tuberculosis. More information on validity and reliability is available in^59^. This parasite stress index correlated negatively (*r* = −0.74, *P* < 0.01) with climatic stress, and negatively (*r* = −0.38, *P* < 0.05) with dopamine genes. Wealth and parasite stress correlated −0.67 (*P* < 0.01).

### Data analysis

We tested our predictions with moderated regression using the linear models function in R from the basic package, using mean-centred variables^60^. We tested whether the interaction between population genetic difference in the dopamine index and climatic stress added any explained variance over and above the main effects. Simple slopes were tested at one SD above and below the mean for climatic stress, using the pequod-package^61^. We used one-sided significance tests in our analysis because of the directionality of our hypothesis. To control for population stratification and genetic clustering, we included continent dummy codes in our analyses^62^.

The descriptive results showing the distribution of the means across samples is presented in Fig. 1. The data was plotted using the rworldmap package in R^63^. The predicted interactions were plotted using the sjplot package^64^ in R, showing the differential effects (and 95% confidence intervals) at minimum and maximum levels of dopaminergic functioning.

## Electronic supplementary material

Data sets
