# Supplementary material for: Dopamine genes are linked to Extraversion and Neuroticism personality traits, but only in demanding climates
Source: Sci Rep. 2018 Jan 29;8:1733. doi: 10.1038/s41598-017-18784-y (PMC5789008; doi:10.1038/s41598-017-18784-y)
Supplement: Supplementary file 1 — Data sets [file 41598_2017_18784_MOESM1_ESM.pdf]

Supplementary files

Dopamine genes are linked to Extraversion and Neuroticism personality traits, but only in demanding climates

Ronald Fischer [a], Anna Lee [a], Machteld N Verzijden [b]

a. School of Psychology, Victoria University of Wellington, New Zealand.

b. Department of Molecular Biology and Genetics - DANDRITE, Aarhus University, Denmark.

Author contributions:

RF designed the research, AL identified marker genes, performed the literature search and created the database, RF analyzed the data, RF & MNV interpreted the results and wrote the paper, RF, AL, & MNV have approved the final version of the paper.

Supplementary Table 1.

Dopamine system index for all nations (including effective sample size), centred climatic demands and a list of countries for which personality data could be matched (listing the effective samples sizes)

	Total N	Dopamine	Climatic demands	N BFI	N NEO-PI-R	N OPQi
Algeria	30	-0.26	0.17	-	-	-
Argentina	156	0.28	0.17	246	-	-
Australia	6988	0.83	0.65	489	-	9120
Austria	322	0.73	0.95	467	444	130
Belgium	48	-0.35	0.78	522	1119	2529
Botswana	148	-0.04	0.26	213	-	-
Brazil	2512	-0.76	-0.77	97	-	1006
Cambodia	98	-1.27	-	-	-	-
Canada	2698	0.23	1.9	1039	848	703
Central African Republic	179	-0.78	-0.9	-	-	-
Chile	6	1.53	0.05	312	-	149
Colombia	89	-2.12	-1.12	-	-	169
Congo	183	-0.69	-0.99	192	-	-
Croatia	32	0.91	0.95	222	722	-
Czech Republic	1068	0.48	1.29	235	570	-
Denmark	165	0.64	0.95	-	1213	6809
Estonia	1135	1.05	1.6	188	1037	-
Ethiopia	133	-0.15	-0.3	240	-	-
Finland	8183	0.31	1.6	122	-	5381
France	2095	1.03	0.61	136	1066	4225
Germany	9475	0.7	0.99	790	2986	1267
Greece	358	1.52	-0.21	-	-	-
Honduras	301	1.1	-1.63	-	-	-
Hong Kong	1257	-1.25	-0.9	201	122	272
Hungary	2603	0.69	0.99	-	312	-
India	5529	-0.1	-0.34	200	214	1098
Iran	509	0.28	0.61	-	-	-
Ireland	994	0.53	0.35	-	-	-
Isreal	1948	0.76	-0.08	394	-	-
Italy	4036	1.01	-0.08	200	623	4550
Ivory Coast	73	-2.47	-1.25	-	-	-
Japan	13543	-0.65	-0.39	259	681	-
Jordan	240	0.73	-0.04	275	-	-
Kenya	421	-0.25	-0.86	-	-	-
Kuweit	12	0.51	-0.26	-	-	-

Laos	117	-0.62	-0.56	-	-	-
Malaysia	276	-0.82	-1.2	141	361	-
Mexico	912	-1.11	-0.51	215	-	149
Micronesia	26	-0.86	-	-	-	-
Mongolia	216	-1.01	2.93	-	-	-
Namibia	56	-0.62	-0.21	-	-	-
Netherlands	4277	0.52	0.69	241	1305	6227
New Zealand	1006	-1.92	-0.34	274	-	2818
Nigeria	306	-1.35	-1.03	-	-	-
Norway	553	0.74	1.21	-	1142	4659
Oman	110	-1.42	-0.51	-	-	-
Pakistan	936	0.63	-0.08	-	-	-
Palestine	51	0.54	-	-	-	-
Papua New Guinea	81	-0.8	-1.33	-	-	-
Paraguay	5	0.7	0.39	-	-	-
Peru	69	-1.34	-1.12	206	439	-
Poland	1694	0.51	1.25	846	-	927
Portugal	93	1.77	-0.43	252	1880	2248
PRC	6384	-0.72	0.91	-	201	3322
Puerto Rico	178	-0.56	-1.46	-	-	-
Russia	6982	0.34	1.72	-	393	-
Samoa	8	-0.59	-1.42	-	-	-
Sierra Leone	22	2.26	-1.5	-	-	-
Singapore	216	0.33	-1.38	-	-	1519
Solomon Islands	22	-0.69	-1.38	-	-	-
Somalia	38	2.08	-1.33	-	-	-
South Africa	383	-0.13	0.09	162	274	4880
South Korea	2479	-0.9	0.78	490	2946	-
Spain	5474	0.71	0.35	273	196	695
Sweden	1860	1.1	1.21	-	720	13244
Switzerland	417	1.1	0.95	214	107	939
Taiwan	3142	-0.72	-0.51	209	544	292
Tanzania	295	-1	-0.77	136	-	-
Tunesia	54	0.11	0.09	-	-	-
Turkey	238	0.87	1.04	412	260	1107
UK	12408	0.67	0.26	483	-	22612
USA	37299	0.2	0.78	2793	1389	4114
Yemen	104	0.39	-1.2	-	-	-

Supplementary Table 2

*** = ALFRED / ^= unpublished**

Study	Nation	Region	Ethnicity	N	DAT1 3' 40bp VNTR			DRD2 Taq1A		DRD2 Taq1B		DRD2 Taq1D		DRD2 rs6275		DRD2 rs6277		DRD2 141C indel		DRD3 ser9gly		DRD4 C521T	
**Study**	**Nation**	**Region**	**Ethnicity**	**N**	**9R**	**10R**	**other**	**A1**	**A2**	**B1**	**B2**	**D1**	**D2**	**C**	**T**	**C**	**T**	**in**	**del**	**ser**	**gly**	**C**	**T**
Aarts et al., 2010	Netherlands		Dutch undergrads	20	22.5	77.5	0																
Aggarwal et al., 2010	South India	Karnataka	Siddi	48				34.1	65.9	29.4	70.6	30.9	69.1										
Aggarwal et al., 2010	South India	Karnataka	Gond	46				25.8	74.2	30.7	69.3	39.2	60.8										
Aggarwal et al., 2010	North India	Gujarat	Varli	42				33.3	66.7	32.1	67.9	30.5	69.5										
Aggarwal et al., 2010	North India	Gujarat	Dangi Konkana	46				32.6	67.4	34.8	65.2	32.2	67.8										
Aggarwal et al., 2010	North India	Gujarat	Kolgha	50				45.9	54.1	47	53	21	79										
Akhmetova et al., 2006*^	Russia		Mari	39	18	82	0																
Akhmetova et al., 2006*^	Russia		Mari	51	16.7	83.3	0																
Akhmetova et al., 2006*^	Russia		Mari	55	17.3	80	2.7																
Akhmetova et al., 2006*^	Russia		Mari	56	24.1	75	0.9																
Akhmetova et al., 2006*^	Russia		Mari	73	11.6	87	1.4																
Akhmetova et al., 2006*^	Russia		Mari	89	15.7	83.1	1.2																
Aklillu et al., 2007	China		Chinese Canadian males*	22				43.2	56.8									75	25				
Al-Eitan et al., 2012	Jordan		Jordanian Arab males	240				15.6	84.4			53.4	46.6	57.1	42.9	54	46	93.3	6.7	63	37		
**Alexander et al., 2011**	**Germany**		**German Caucasian males**	**100**	**21.5**	**74.5**	**4**																
Alfimova et al., 2007	Russia		Russian	115																		47.35	52.65
Alvarez et al., 2010	Spain		Spanish Caucasian bone injury	290	30.9	67.3	1.7																
**Amadeo et al., 1993**	**France**		**SE France**	**20**				**5**	**95**														
Amorim et al., 2011	Brazil		Afro-Brazilian Mocambo	76				43.2	56.8	28.4	71.6	28.4	71.6										
Amorim et al., 2011	Brazil		Afro-Brazilian Rio das Ras	91				38.5	61.5	41.7	58.3	41.7	58.3										
Amorim et al., 2011	Brazil		Afro-Brazilian Sacutiaba	30				23.4	76.6	40.8	56.6	40.8	56.6										
**Anghelescu et al., 2010**	**Germany**		**German Caucasian**	**144**	**30.9**	**67.1**	**2**																
Arinami et al., 1997	Japan		Japanese	312														78.5	21.5				
Arinami et al., 1999	Japan		male Japanese	125														84	16				
Ariza et al., 2012	Spain		Spanish	42				15.5	84.5														
Asherson et al., 1996	UK		Welsh	100																67	33		
Audrain-McGovern et al., 2004	US Euro		US Euro	615	27.5	72.5	0	18	82														
Azzato et al., 2009	US Euro		US Euro (89%), Afro(4.1%), other(6.9%)	887	27.85	72.15	0																
Bagozzi et al., 2012	US mixed		US salespeople	65				28.5	71.5														
Balci et al., 2012	US mixed		US Penn Uni students	30	30	70	0	28.4	71.6														
Ball et al., 1998	US Euro		US Caucasion 6-15yo children	51	20.5	79.5	0	25.5	74.5											73.5	26.5		
Banoei et al., 2008b	Iran		Armenians	50	50	50	0																
Barkley et al., 2006	US Euro		US Caucasian	61	21.3	78.7	0																
Barr & Kidd 1993	Brazil		Surui	88				56	44														
Barr & Kidd 1993	Brazil		Karitiana	98				60	40														
Barr & Kidd 1993	Cambodia		US Cambodian*	50				42	58														
Barr & Kidd 1993	Central African Republic		CAR Pygmy	62				24	76														
Barr & Kidd 1993	China		Chinese	64				47	53														
Barr & Kidd 1993	Colombia		Colombian	26				65	35														
Barr & Kidd 1993	North India	Assam	Asian Assamese	18				33	67														
Barr & Kidd 1993	South India	Andhra Pradesh	Thoti	24				58	42														
Barr & Kidd 1993	Israel		Druze	28				11	89														
Barr & Kidd 1993	Italy		Roman Jews*	40				17	83														
Barr & Kidd 1993	Japan		US Japanese*	84				48	52														
Barr & Kidd 1993	Mexico		Mayan	70				70	30														
Barr & Kidd 1993	Peru		Quechua	34				53	47														
Barr & Kidd 1993	US Amerindian		American Muskoke	20				75	25														
Barr & Kidd 1993	Yemen		Yemenite Jews*	22				9	91														
Barr & Kidd 1993	Zaire		Zaire Pygmy	34				32	68														
Basu et al., 2003*	North India	West Bengal	Ho	48				56.7	43.3	50	50	16.7	83.3										
Basu et al., 2003*	South India	Kerala	Irula	50				36.7	63.3	38.8	61.2	43	57										
Basu et al., 2003*	South India	Kerala	Kota	45				51.1	48.9	23.3	76.7	26.7	73.3										
Basu et al., 2003*	South India	Kerala	Kurumba	54				46.3	53.7	33.3	66.7	50	50										
Basu et al., 2003*	North India	Mizoram	Mizo	26						25	75	1.7	98.3										
Basu et al., 2003*	South India	Kerala	Toda	50				20.2	79.8	9.1	90.9	56.1	43.9										
Basu et al., 2003*	North India	West Bengal	Toto	30				63.3	36.7	61.7	38.3	35	65										
Batra et al., 2000	Germany		German	60				21.55	78.45														
Bau et al., 2000	Brazil		Brazilian Caucasians	114				21.1	79														
Beaver et al., 2007b	US mixed		US male youths	872				24.5	75.5														
Beaver et al., 2008b	US mixed		US youths	1816	22.85	77.15	0																
Becker et al., 2008	Germany		German Caucasian children	305	25.9	74.1	0																

Study	Nation	Region	Ethnicity	N	9R	10R	other	A1	A2	B1	B2	D1	D2	C	T	C	T	in	del	ser	gly	C	T
Behravan et al., 2008	Iran		Iranian	63				35.75	64.25	19.05	80.95												
Bergen et al., 2005	US Euro		Euro-American females	84				23	77					76.5	23.5	58.5	41.5	92.5	7.5				
Berggren et al., 2006	Sweden		Swedish Caucasians	842				17.6	82.4														
Berlin et al., 2000	France		French Caucasian	202				21.1	78.9														
Berman et al., 1995	US Euro		US 10-14yo boys, 74% Caucasian	182				25.3	74.7														
Berman et al., 2009	US Euro		Euro-American males	12				25	75														
Bermisheva et al., 2007*	Russia		Udmurt	29	12.1	82.7	5.2																
Bermisheva et al., 2007*	Russia		Udmurt	36	18.1	75	6.9																
Bermisheva et al., 2007*	Russia		Udmurt	54	9.2	86.1	4.7																
Bermisheva et al., 2007*	Russia		Udmurt	56	11.6	84.8	3.6																
Bermisheva et al., 2007*	Russia		Udmurt	79	12	86.1	1.9																
Bermisheva et al., 2007*	Russia		Udmurt	122	16	82	2																
Berrettini et al., 1996	US Afro		Afro-Americans	88				42.5	57.5	22.5	77.5												
Bertolino et al., 2006	Italy		Italian	62	35.5	64.5	0																
Bertolino et al., 2008	Italy		Italian	82	34.15	65.85	0																
Bhaskar et al., 2009	South India	Kerala	Adiyan	42	13.1	84.5	2.4																
Bhaskar et al., 2009	South India	Andhra Pradesh	Agnikula Kashtriya	38	7.9	90.8	1.3																
Bhaskar et al., 2009	South India	Tamil Nadu	Badaga	64	21.1	73.4	5.5																
Bhaskar et al., 2009	South India	Andhra Pradesh	Gandla	25	14	84	2																
Bhaskar et al., 2009	North India	Maharastra	Korku	31	17.7	80.6	1.7																
Bhaskar et al., 2009	South India	Tamil Nadu	Kota	54	24.1	75.9	0																
Bhaskar et al., 2009	South India	Kerala	Kurumba	34	13.2	86.8	0																
Bhaskar et al., 2009	South India	Tamil Nadu	Lingayat	36	25	72.2	2.8																
Bhaskar et al., 2009	South India	Kerala	Muduga	48	7.3	84.5	8.2																
Bhaskar et al., 2009	North India	Madhya Pradesh	Ojha	25	24	74	2																
Bhaskar et al., 2009	North India	Madhya Pradesh	Rajgond	35	5.7	94.3	0																
Bhaskar et al., 2009	North India	Madhya Pradesh	Sonr	39	11.5	85.9	2.6																
Bhaskar et al., 2010	South India	Tamil Nadu	Kota	115				47.35	52.55	27.4	72.6	35.6	64.3										
Bhaskar et al., 2012	South India	Tamil Nadu	Kota	95	32.6	67.4	0																
Bhaskar et al., 2012	South India	Tamil Nadu	Badaga	61	23	71.3	5.7																
Bhowmik et al., 2011	North India	West Bengal	Indian	175	14.7	83.6	1.7															46.3	53.7
Billett et al., 1998	Canada		Canadian	103	19	81	0	22.5	77.5											62.5	37.5		
Birkas et al., 2006	Hungary		Hungarian	57																		52.6	48.4
Blair et al., 2008	US Euro		US Caucasian	121																53.5	46.5		
Blair et al., 2008	US Euro		US Caucasian	151																41	59		
Blanchard et al., 2011	UK		UK	254	24.6	75.4	0																
Bloch et al., 2009	US Afro		US Afro	257																27.1	72.9		
Blum et al., 1990	US Afro		US Afro-American	11				27	73														
Blum et al., 1990	US Euro		US Euro-American	24				17	83														
Blum et al., 1996	US Euro		US Caucasian	20				0	100														
Bolos et al., 1990	US Euro		US Caucasian	65				15.75	84.25														
Bolton et al., 2010	Scotland		Scottish	2022				21.05	78.95							46.85	53.25						
Boutwell & Beaver 2008	US Euro		US adolescents	1352				75	25														
Breen et al., 1999	Scotland		Scottish	292														88	12				
Breen et al., 1999	UK		English	145														90	10				
Brissenden et al., 2012*^	Mongolia		Mongolian	66						32.6	67.4			43.4	56.6	88.7	11.3						
Brissenden et al., 2012*^	Mongolia		Tsaatan	50										33	67	97	3						
Brissenden et al., 2012*^	Russia		Tsaatan	48						30.2	69.8												
Brookes et al., 2007	UK		UK	7	35.65	64.35	0																
Bruins-Slot et al., 1998	France		French Caucasian	42																73.85	26.15		
Burt & Mikolajewski 2008	US Euro		Euro-American males	197	30	70.1	0																
Camara et al., 2010	Spain		Spanish	632																		46.95	52.95
Campa et al., 2007	Norway		Norwegian Caucasian	222				83.8	16.2	83.9	16					55.8	44.1	98.05	1.95			68	32
Caprini et al., 2011	Scandinavian mixed		Danish/Swedish/Norwegian	1468								60.85	39.15										
Catalano et al., 1994	Italy		Italian	97																57.25	42.75		
Cerasa et al., 2009	Italy		Italian Caucasian	70				10.7	89.3														
Chakrabarti et al., 2002*	North India	Mizoram	Mizo	28				26.8	73.2			26.8	73.2										
Chakrabarti et al., 2002*	North India	Uttar Pradesh	Katharia Tharu	37				36.5	63.5	36.5	63.5	28.4	71.6										
Chakrabarti et al., 2002*	North India	Uttar Pradesh	Rana Tharu	54				47.2	52.8	43.5	56.5	36.2	64.8										
Chakraborty et al., 2008	US mixed		US	80				47.55	52.45	16.25	83.75	21.05	78.95							75.25	24.65		
Chang et al., 1996*	Mexico		Maya	45	6.7	92.3	1	70.8	29.2	71.4	28.6	2.8	97.2			98	2	83.3	16.7	54.7	45.3	13.7	86.3
Chang et al., 1996*	Brazil		Karitiana	51	0	100	0	59.4	40.6	62.3	37.7	0	100			100	0			40	60	4.9	95.1
Chang et al., 1996*	Cambodia		US Cambodians*	23	20	80	0			45.7	54.3					95.8	4.2			68	32	26.1	73.9

Study	Nation	Region	Ethnicity	N	9R	10R	other	A1	A2	B1	B2	D1	D2	C	T	C	T	in	del	ser	gly	C	T
Chang et al., 1996*	Central African Republic		Biaka	62	22.3	56.2	21.5	26.8	73.2	5.9	94.1	15	85	16	84	99.3	0.7	68.6	31.4	37.9	62.1	34.8	65.2
Chang et al., 1996*	China		US Han Chinese*	49	6	91.4	2.6	44.8	55.2	44.7	55.3	6	94	46	54	95.7	4.3	87.3	12.7	66.1	33.9	35.3	64.7
Chang et al., 1996*	Colombia		Guihiba	10						60	40	0	100	62.5	37.5	100	0			25	75		
Chang et al., 1996*	Denmark		Danish blood-bank donors	32	21.6	76.5	1.9			13	87			68	32	42.2	57.8					45.7	54.3
Chang et al., 1996*	DR Congo		Mbuti	20	28.2	35.9	35.9	32.1	67.9	0	100	15.4	84.6	23	77	96	4.1	66.2	33.8	59	41	47	53
Chang et al., 1996*	Finland		Finnish	33	10	90	0	22.2	77.8	25.8	74.2	51.5	48.5	62	38	58.8	41.2			68.6	31.4	40	60
Chang et al., 1996*	North India	Assam	Kachari	14						35.7	64.3	27.8	72.2	61.8	38.2	73.5	26.5			73.5	26.5		
Chang et al., 1996*	South India	Andhra Pradesh	Thoti	12						58.3	41.7			73.1	26.9	85.7	14.3			45.8	54.2		
Chang et al., 1996*	Israel		Druze	70														97.9	2.1			40.7	59.3
Chang et al., 1996*	Italy		Sephardic Jews*	27												42.6	57.4						
Chang et al., 1996*	Japan		US Japanese*	44	1.1	97.7	1.2			44.4	55.6			52	48	94.1	5.9	76.5	23.5	69.6	30.4	46.8	53.2
Chang et al., 1996*	Malaysia		Orang Asli/Malay	6						50	50			45.5	54.6	100	0			72.7	27.3		
Chang et al., 1996*	Micronesia		Micronesians	26	13.5	86.5	0	29.4	70.6	27.3	72.7	18.9	81.1	43	57	87.1	12.9			59.5	40.5	27.8	72.2
Chang et al., 1996*	Papua New Guinea		Papua New Guinean	19						2.6	97.4			77.5	22.5	81	19.1			63.2	36.8		
Chang et al., 1996*	Peru		Quechua	15				59	41	55.6	44.4	0	100	61	39	96.7	4.4			35	65		
Chang et al., 1996*	Russia		Adygei	52	23.1	74.1	2.8	16	84	11.3	88.7	62.3	37.7	70	30	44.3	55.7			72.2	27.8	43.3	56.7
Chang et al., 1996*	Russia		Yakut	25	4.9	94.1	1	29.4	70.6	32	68			34	66	94.1	5.9	94.4	5.6	92	8	30.4	69.6
Chang et al., 1996*	Samoa		Samoans	8						50	50			61.1	38.9	83.3	16.7			50	50		
Chang et al., 1996*	Solomon Islands		Nasioi Melanesians	22																		43.2	56.8
Chang et al., 1996*	Spain		Sephardic Jews*	27																72.2	27.8		
Chang et al., 1996*	Taiwan		Ami	38	13.2	86.8	0	32.5	67.5	35.9	64.1	5	95	39	61	97.5	2.5			78.8	21.2	32.5	67.5
Chang et al., 1996*	Taiwan		Atayal	28	6	94	0	54.8	45.2	58.5	41.5	0	100	57	43	100	0			81	19	16.3	83.8
Chang et al., 1996*	Taiwan		Han Chinese	42	10.4	84.4	5.2	40.8	59.2	37.8	62.2	10	90	45	55	91.7	8.3			64.3	35.7	32.6	67.4
Chang et al., 1996*	US Amerindian		Cheyenne	48	1.8	98.2	0			75	25			83	17	92.9	7.1			68.8	31.2	26.4	73.6
Chang et al., 1996*	US Amerindian		Maya	52										69	31								
Chang et al., 1996*	US Amerindian		Muscogee	10						70	30			90	10	80	20			85	15		
Chang et al., 1996*	US Amerindian		Pima	35	0	100	0	64.3	35.7	61.5	38.5					100	0			41.2	58.8	6	94
Chang et al., 1996*	US Amerindian		SW Amerindian	43	1.2	98.8	0			70	30												
Chang et al., 1996*	US Asian		Cambodian	24										46	54								
Chang et al., 1996*	Yemen		Yemenite Jews*	39	48.8	43.8	7.4	8.1	91.9	5.1	94.9	52.3	47.7	44	56					70.9	29.1	41.9	58.1
Chen et al., 1996	Taiwan		Atayal	41				39	61														
Chen et al., 1997a*^/1997b/2001	Taiwan		Han Chinese	59	8	92	0	37.15	62.85					34	66			89	11				
Chen et al., 1997a*^/1997b/2001	Taiwan		Paiwan	34	4.5	95.5	0	37.1	62.8					53	47			100	0				
Chen et al., 1997a*^/2001	Taiwan		Ami	23	11	89	0	23.9	76.1					35	65			93.5	6.5				
Chen et al., 1997a*^/2001	Taiwan		Atayal	30	8	92	0	58.05	41.95					55	45			93.5	6.5				
Chen et al., 1997c	Taiwan		Han Chinese	100																70	30		
Chen et al., 2001	Taiwan		Bunun	56	15.5	84.5	0	35.3	64.7					32	68			97.5	2.5				
Chen et al., 2012b	US Euro		US Caucasian	105				33.4	66.6														
Chiaroni et al., 2000	France		French Caucasian	60																67.55	32.45		
Choudhry et al., 2006	Puerto Rico		Puerto Rican	178								45	55										
Choudhry et al., 2006	US Hispanic		US Mexican	181								35	65										
Cicchetti et al., 2011	US mixed		US 73% 'minority ethnic'	47																		31.9	68.1
Clague et al., 2010	US Euro		US Caucasian	557				19.85	80.15	16.85	83.15												
Colzato et al., 2010	Netherlands		Dutch Caucasian	128												51.6	48.4						
Colzato et al., 2011	Netherlands		Dutch Caucasian	154												48	52						
Comings et al., 1991	US Euro		US Caucasian	108				11.1	88.9														
Comings et al., 1996c	US mixed		US	67				15.7	84.3														
Comings et al., 1997	US Euro		US Caucasian	136				17.75	82.25														
Comings et al., 1999a	US Euro		US Caucasian	305																69.05	30.95		
Comings et al., 2003	US Euro		US Caucasian females	145				18.65	81.35														
Cook et al., 1992	US Euro		US Caucasian males	20				15	85														
Cordeiro et al., 2009	Brazil		Brazilian	733														81.95	18.05				
Costa-Mallen et al., 2000	US Euro		US Caucasian	231						13.6	86.4												
Costa-Mallen et al., 2005	US Euro		US Caucasian	202				18.6	81.4														
Crettol et al., 2008	Switzerland		Swiss Caucasian	217				16.15	83.85							40.35	59.65						
Crocq et al., 1996	DR Congo		Congolese	56																11.6	88.4		
Crocq et al., 1996	France		Alsatian Caucasian	101																66.75	33.15		
Cruz et al., 1995	Mexico		Mexican	38				53.95	46.05														
Das et al., 2011c	North India	West Bengal	Indo-Caucasoid	96	16	81.7	2.3																
David et al., 2010	US Afro		Afro-American	173				29.35	70.65							17.55	82.45	60.75	39.25				
David et al., 2010	US Euro		Euro-American	376				22.5	77.5							55	45	83.25	16.75				
Davis et al., 2008	Canada		Canadian Caucasian	59				17.8	82.2			47.5	52.5			61	39	82.2	17.8	63.95	36.05		
de Brettes et al., 1998	France		French Caucasian	204				21.1	78.9														

Study	Nation	Region	Ethnicity	N	9R	10R	other	A1	A2	B1	B2	D1	D2	C	T	C	T	in	del	ser	gly	C	T
de los Cobos et al., 2007	Spain		Spanish Caucasian	145				17.6	82.4														
Demiralp et al., 2007	Turkey		Turkish males	48	29	69	2																
Dikeos et al., 1999	Greece		Greek	38																71.05	28.95		
Dmitrzak-Węglarz et al., 2005	Poland		Polish	119	30.1	69.9	0																
Dobashi et al., 1997	Japan		Japanese	100										48	52								
Doehring et al., 2009a	Germany		German Caucasian	300				17.5	82.5	13.8	86.2			67	33	47	53	85.8	14.2				
Doehring et al., 2009b	Germany		German Caucasian	99				14.1	85.9					68.7	31.3	44.4	55.6	82.3	17.7				
Domínguez et al., 2007	Spain		Spanish	354																67.8	32.2		
Doucette-Stamm et al., 1995	US Afro		Afro-American	457	17.2	72.9	9.9																
Doucette-Stamm et al., 1995	US Euro		Euro-American	467	27.1	71.9	1																
Doucette-Stamm et al., 1995	US Hispanic		Hispanic American	150	25.9	70.9	3.2																
Drtilkova et al., 2008	Czech Republic		Czech boys	153				15	85														
Du & Wan 2009/Du et al., 2010/2011	US Hispanic		Mexican-American	338	15.9	82.2	1.9											83.85	16.15				
Duaux et al., 1998	France		French Caucasian males	70																70.75	29.25		
Dubertret et al., 2001	France		French	50				27	73	20	80												
Dubertret et al., 2004	France		French	83				40	60	35	65	62	38					72	28				
Ducci et al., 2011	Finland		Finnish	4762				17	83														
Duran-Gonzalez et al., 2011	US Hispanic		Mexican-American	317				38.45	61.55														
Durany et al., 1996	Spain		Spanish	100																66.5	33.5		
Ebstein et al., 1996d	Israel		73% Ashkenazi Jews*	124																71.4	28.6		
Ebstein et al., 1997d	Israel		Ashkenazi Jews*	109																75.7	24.3		
Ebstein et al., 1997d	Israel		non-Ashkenazi Jews*	136																70.35	29.65		
Ebstein et al., 1997d	Italy		Italian	27																73.85	26.15		
Eisenberg et al., 2007	US mixed		US students 44% Euro	193				29.75	70.25														
Eisenberg et al., 2008	Kenya		Ariaal	156				28.25	71.85														
Emanuele et al., 2007	Italy		Italian	349				29.7	70.3	33.4	66.6												
Eny et al., 2009	Canada		Canadian Euro	313												49	51						
Eny et al., 2009	Canada		Canadian East Asian	245												94.5	5.5						
Eny et al., 2009	Canada		Canadian South Asian	81												65	35						
Eny et al., 2009	Canada		Canadian other	50												74	26						
Ettinger et al., 2006	Canada		Canadian Caucasian males	31	22.6	77.4	0													62.95	37.05		
Fan et al., 2010	China		Han Chinese	403												95.5	4.4						
Fathalli et al., 2008	Canada		Canadian	118																71.65	28.35		
Fathalli et al., 2008	Tunisia		Tunisian	54																56.5	43.5		
Feher et al., 2011	Hungary		Hungarian	189																72.8	27.2		
Feher et al., 2011	Hungary		Roma	189																69.35	30.65		
Felten et al., 2012	Germany		German	211												45	55						
Fernàndez-Castillo et al., 2010	Spain		Spanish Caucasian	169				16.6	83.4	9.45	90.55												
Filonzi et al., 2009	Italy		Italian Caucasian	150	27.35	63.35	4.7																
Filopanti et al., 2008	Italy		Italian	212				15.5	84.5	12.75	87.25												
Finckh et al., 1996b	Germany		German Caucasian	131				16.05	83.95														
Flegontova et al., 2009*	Russia		Adygei	48				15.3	84.7														
Flegontova et al., 2009*	Russia		Adygei	98						11.7	88.3	63.3	36.7										
Flegontova et al., 2009*	Russia		Belarusian	70				14.3	85.7	12.9	87.1	55	45										
Flegontova et al., 2009*	Russia		Belarusian	75				12	88	11.3	88.7	51.3	48.7										
Flegontova et al., 2009*	Russia		Belarusian	85				16.5	83.5	15.3	84.7	60	40										
Flegontova et al., 2009*	Russia		Kalmyks	104				34.6	65.4	35.6	64.4	14.4	85.6										
Flegontova et al., 2009*	Russia		Khanty	62				25.8	74.2	23.4	76.6	25	75										
Flegontova et al., 2009*	Russia		Komi-Zyrian	109				22.5	77.5	20.6	79.4	51.8	48.2										
Flegontova et al., 2009*	Russia		Komi-Zyrian	112				15.2	84.8	13.8	86.2	58.9	41.1										
Flegontova et al., 2009*	Russia		Russians	65				15.4	84.6	13.1	86.9	55.4	44.6										
Flegontova et al., 2009*	Russia		Russians	71				23.2	76.8	21.1	78.9	42.3	57.7										
Flegontova et al., 2009*	Russia		Russians	85				18.9	81.1	15.8	84.2	47.4	52.6										
Flegontova et al., 2009*	Russia		Russians	109				26.1	73.9	25.2	74.8	53.7	46.3										
Flegontova et al., 2009*	Russia		Russians	117				15.4	84.6	14.5	85.5	56	44										
Flegontova et al., 2009*	Russia		Russians	147				15.6	84.4	15.3	84.7	44.6	55.4										
Flegontova et al., 2009*	Russia		Yakut	118				21.4	78.6	26.9	73.1	6.8	93.2										
Foley & Dodd 1996	Australia		Australian Caucasian	50				20	80														
Foley et al., 2004	Australia		Australian Caucasian	43	30.5	69.5	0	22	78	22	78												
Forbes et al., 2009	US mixed		US	76														88.15	11.85				
Fossella et al., 2002	US mixed		US and Chinese	200	23	75	2															43	57
Fossella et al., 2006a	US mixed		US	15				16.7	83.3														
Frank et al., 2007	US mixed		US 70% Caucasian	69												46.35	53.65						

Study	Nation	Region	Ethnicity	N	9R	10R	other	A1	A2	B1	B2	D1	D2	C	T	C	T	in	del	ser	gly	C	T
Franke et al., 2008	Netherlands		Dutch Caucasian	528	23.15	76.85	0																
Freire et al., 2006	Brazil		Brazilian Euro	112				17.45	82.55														
Furlong et al., 1998	US Euro		UK 97% Caucasian	262														89.7	10.3				
Gabriela et al., 2009	Mexico		Mexican adolescents	84	14.6	85.4	0																
Gaitonde et al., 1996	UK		UK Caucasian	77																65.65	34.35		
Galeeva et al., 2001*^	Russia		Bashkir	70	15	82	3																
Galeeva et al., 2001*^	Russia		Chuvash	56	12	86	2																
Galeeva et al., 2001*^	Russia		Mari	56	19	80	1																
Galeeva et al., 2001*^	Russia		Tatar	56	19	81	0																
Galeeva et al., 2001*^	Russia		Udmurt	64	16	81	3																
Galeeva et al., 2001*^	Russia		Komi-Zyrian	50	22	75	3																
Galeeva et al., 2001*^	Russia		Russians	65	15	83	2																
Garcia-Martin et al., 2010	Spain		Spanish Caucasian	282																76.25	23.75		
Gauniyal et al., 2011	South India	Karnataka	Siddi	34				34	66	29.4	70.6	30.9	69.1										
Geijer et al., 1994	Sweden		Swedish	65				21	79	19.1	80.9												
Gelernter & Kranzler 1999a	US Euro		US Euro-American	136				17.65	82.35	15.45	84.55	62.5	37.5					93.7	6.3				
Gelernter et al., 1991	US Euro		US Caucasian	199				19.85	80.15														
Gelernter et al., 1998a	Japan		Japanese	48				35	65			10	90					79	21				
Gelernter et al., 1998a	US Afro		Afro-American	42				36	64			26	74					61	39				
Gelernter et al., 1998a	US Euro		Euro-American	83														89	11				
Gelernter et al., 1998b	Brazil		Surui	28	0	100	0													43	57		
Gelernter et al., 1998b	China		Chinese	47	6	92	2													62	38		
Gelernter et al., 1998b	DR Congo		Mbuti	36	28	37	35													58	42		
Gelernter et al., 1998b	Mexico		Maya	41	6	93	1													56	44		
Gelernter et al., 1998b	Papua New Guinea		Nasioi	23	0	93	7													50	50		
Gelernter et al., 1998b	Russia		Adygei	50	21	77	2													74	26		
Gelernter et al., 1998b	US Afro		Afro-American	39	15	76	9													29	71		
Gelernter et al., 1998b	US Euro		Euro-American	61	28	71	1	18.95	81.05	16	84	61	39							75	25		
Gemignani et al., 2005	Spain		Spanish	270				16.7	83.3	11	89					62.5	37.5	95.65	4.35				
Gervai et al., 2005	Hungary		Hungarian Caucasian	185																		49.7	50.3
Ghosh et al., 2013	North India	Lucknow	Indian	200				32	68					52.75	47.25			89	11				
Gillath et al., 2008	US mixed		US 44% Asian, 31% Caucasian	147				31.65	68.35														
Goldman et al., 1993b	US Afro		US Afro-American	43				37.25	62.75														
Goldman et al., 1993b	US Amerindian		Jemez Pueblo	23				63.05	36.95														
Goldman et al., 1993b	US Amerindian		Cheyenne	52				79.85	20.15														
Gómez-Casero et al., 1996	Spain		Spanish Caucasian	53	34.05	60.45	2.8													66	34		
Gong et al., 2010	China		Han Chinese	482	0	93	7	39.7	60.3	42.05	57.95									71.5	28.5	43.9	56.1
Gorwood et al., 2000a	France		French	35				42.9	57.1														
Gorwood et al., 2000b	France		French Caucasian	49				40.8	59.2														
Goudreau et al., 2002	US Euro		US 97.5% Euro	146	8.2	54.1																	
Grevle et al., 2000	Norway		Norwegian	81				12	88														
Griffon et al., 1996	France		Alsacians	85																66	34		
Guo & Tillman 2009	US mixed		US 59% White, 17% Black, 14.6% Hispanic, 8.8%Asian	2286				26.5	73.5														
Gupta et al., 2009	South India	Bangalore	Southern Indian	223												60.45	39.55	87	13				
Güzey et al., 2011	Norway		Norwegian	250	19	80	1	21	79											68	32		
Hallikainen et al., 2003	Finland		Finnish Caucasian males	884				23.9	76.1														
Han et al., 2007	South Korea		South Korean adolescent males	75				34.65	65.35														
Han et al., 2008	South Korea		South Korean	40				30	70														
Hanninen et al., 2006	Finland		Finnish	384												50	50						
Hansen et al., 2005	US mixed		US 2/3 AA, 1/3 EA	24										62.5	37.5	60.4	39.6						
Hawi et al., 1998	Ireland		Irish	235																64.5	35.5		
Heinz et al., 2000	US mixed		US no ethnicity data	11	13.65	86.35	0																
Hemmings et al., 2006	South Africa		South African Caucasian	121																		55.8	44.2
Henderson et al., 2000	Australia		Australian Caucasian	862																66.95	33.05		
Henderson et al., 2000	Australia		Australian Caucasian	1465																67.2	32.9		
Hietala et al., 1997	Finland		Finnish males	50				11	89														
Higuchi et al., 1994 / 1996	Japan		Japanese	200																69.5	30.5		
Higuchi et al., 1995	Japan		Japanese	70	5.7	89.3	5													74.3	0		
Himei et al.,, 2002*	Japan		Japanese	103														82	18				
Hirvonen et al., 2009a	Finland		Finnish	38				14.45	85.55							48.65	51.35	90.8	9.2				
Ho et al., 2008	Hong Kong		HK Chinese males	165																		40.35	59.75
Hoenicka et al., 2006	Spain		Spanish	364												36.5	63.5						

Study	Nation	Region	Ethnicity	N	9R	10R	other	A1	A2	B1	B2	D1	D2	C	T	C	T	in	del	ser	gly	C	T
Holmboe et al., 2010	UK		UK Caucasian	122				17.75	82.25														
Hong et al., 2003	Taiwan		Taiwanese Chinese males	109	26.3	72.6	1.1																
Hori et al., 2001	Japan		Japanese	201														94.5	5.5				
Hou et al., 2009	China		Xi'an Han Chinese	492	4.4	91.2	4.3	39.95	60.05														
Hsieh et al., 2009	Taiwan		Chinese females	112										54	46								
Hu et al., 1999*^	Russia		Mordvin	58	29	69	2																
Hu et al., 2013	Taiwan		Han Chinese males	328				34.75	65.25														
Huang et al., 2012	Taiwan		Han Chinese	255				35.9	64.1														
Huuhka et al., 2008	Finland		Finnish Caucasian	383												59.275	13.575						
Inada et al., 1995	Japan		Japanese	48																78	21.9		
Inada et al., 1996	Japan		Japanese	117	7.7	90.2	2.1																
International HapMap Consortium 2003*^	China		Han Chinese	84						44	56												
International HapMap Consortium 2003*^	China		US Han Chinese*	85						48.2	51.8												
International HapMap Consortium 2003*^	North India	Gujarat	Gujarati	88						24.4	75.6												
International HapMap Consortium 2003*^	Italy		Italians	88						13.1	86.9												
International HapMap Consortium 2003*^	Japan		Japanese	86						40	60												
International HapMap Consortium 2003*^	Kenya		Luhya	90						13.3	86.7												
International HapMap Consortium 2003*^	Kenya		Masai	143						14	86												
International HapMap Consortium 2003*^	Nigeria		Yoruba	113						17.7	82.3												
International HapMap Consortium 2003*^	US Afro		Afro-Americans	53						18.9	81.1												
International HapMap Consortium 2003*^	US Euro		US Europeans	113						12.4	87.6												
International HapMap Consortium 2003*^	US Hispanic		Hispanic American	50						35	65												
Ishiguro et al., 1998	Japan		Japanese	152				35	65									76.5	23.5				
Ishiguro et al., 2000b	Japan		Japanese	269																		40.5	59.5
Ishii et al., 2012a	Japan		Japanese	119										50	50								
Johann et al., 2005	Germany		German	232														90.5	9.5				
Jones & Peroutka 1998	US Euro		US Caucasian	116				16.8	83.2	10.75	89.25												
Jönsson et al., 1998b / 1999a / 1999b	Sweden		Swedish Caucasian	56				17.8	82.2	19.55	80.35							87	13	73.5	26.5	43	57
Jönsson et al., 2001 / 2003b / 2003c	Sweden		Swedish	236																70.05	29.95	42	58
Joober et al., 2000	Canada		Canadian Caucasian	89	25.25	74.75	0													71.35	28.65		
Kahsar-Miller et al., 1999	US Euro		US Caucasian females	96																66.1	33.9		
Kampman et al., 2003	Finland		Finnish	94														96.8	3.2				
Kang et al., 1999*	Ethiopia		Ethiopian Jews*	16	18.8	79.7	1.5	7.8	92.2	0	100	34.4	65.6	31	69	75	25			40.3	59.7	53.2	46.8
Kang et al., 1999*	Ireland		Irish	92	30.4	69.1	0.5	21.6	78.4	14.4	85.6	60.8	39.2	68	32	45.1	54.9	88	12	64.8	35.2	34.8	65.2
Kang et al., 1999*	Nigeria		Yoruba	57	13.3	73.5	13.2	47.4	52.6	15.8	84.2	15.4	84.6	33	67	96.8	3.3	27.2	72.8	20.8	79.2	39.3	60.7
Kang et al., 1999*	Russia		Russians	46	19.6	80.4	0	17.7	82.3	16.3	83.7	52.1	47.9	68	32	45.7	54.3			55.2	44.8	18.8	81.3
Kang et al., 1999*	Taiwan		Hakka	34	8.8	86.8	4.4	47.5	52.5	48.8	51.2	4.9	95.1	52	48	93.9	6.1			75.6	24.4	43.4	56.6
Kang et al., 1999*	Yemen		Yemenite Jews*	43												60.5	39.5						
Kang et al., 2011	Brazil		Brazilian	50										64	36	67	11						
Kang et al., 2011	US Euro		US European	451												63.25	15.75						
Kasiakogia-Worlley et al., 2011	UK		UK	917				20.4	79.6							55.4	44.6						
Katsuragi et al., 2001	Japan		Japanese	105														74.8	25.2				
Kazantseva et al., 2011	Russia		Russians	233	22.5	77.5	0	21.85	78.15					66	34								
Kazantseva et al., 2011	Russia		Tatars	419	20.55	79.45	0	23.25	76.75					59.2	40.8								
Keeling et al., 2009	US mixed		US no ethnicity data	408																68.1	31.9		
Kelada et al., 2002	US Euro		US Caucasian	290						14.8	36.05												
Kelada et al., 2006	US Euro		Euro-American	395	25.45	74.55	0																
Kennedy et al., 1995	Italy		Italian	64																73.5	26.5		
Kennedy et al., 1995	US mixed		US	188																68.5	31.5		
Kereszturi et al., 2007	Hungary		Hungarian Caucasian	284																		43.85	56.15
Kéri et al., 2005	Hungary		Hungarian	60																66.7	33.3		
Khusnutdinova et al., 2003*^	Russia		Chuvash	47	14.5	80.3	5.2																
Khusnutdinova et al., 2003*^	Russia		Chuvash	87	26.3	73	0.7																
Khusnutdinova et al., 2003*^	Russia		Chuvash	95	21.2	74.4	4.4																
Khusnutdinova et al., 2003*^	Russia		Chuvash	108	10.8	84.4	4.8																
Kidd et al., 1996	Brazil		Ticuna	67								20	80										
Kidd et al., 1996	Cambodia		US Cambodian*	25								4	96										
Kidd et al., 1996	Japan		US Japanese*	49								5	95										
Kidd et al., 1996	Papua New Guinea		Melanesian Nasioi	22								26	74										
Kidd et al., 1996	Russia		Yakut	46								6	94										
Kidd et al., 1996	US Amerindian		Cheyenne	56								6	94										
Kidd et al., 1996	US Amerindian		Jemez Pueblo	44								0	100										

Study	Nation	Region	Ethnicity	N	9R	10R	other	A1	A2	B1	B2	D1	D2	C	T	C	T	in	del	ser	gly	C	T
Kidd et al., 1996	US Amerindian		Pima	52								0	100										
Kidd et al., 1998*	Botswana		San	49				39.8	60.2			19.4	80.6										
Kidd et al., 1998*	Botswana		San	50				35	65			21	79										
Kidd et al., 1998*	Botswana		San	49						22.4	77.6												
Kidd et al., 1998*	Namibia		San	50						10	90												
Kidd et al., 1998*	South Africa		Sotho	50				48	52			10	90										
Kidd et al., 1998*	South Africa		Tsonga	50				39	61			14	86										
Kidd et al., 1998*	South Africa		Sotho	50						23	77												
Kidd et al., 1998*	South Africa		Tsonga	50						10	90												
Kidd et al., 1998*	South Korea		South Koreans	127						37.4	62.6												
Kidd unpublished*^	Denmark		Danish blood donors	90														92.8	7.2				
Kidd unpublished*^	Hungary		Hungarians	87						13.1	86.9			70.7	29.3					80.1	19.9		
Kidd unpublished*^	South India	Kerala	Keralite	14				50	50														
Kidd unpublished*^	South India	Kerala	US Keralite*	15						26.8	73.2	37	63	70	30	58.3	41.7			53	47		
Kidd unpublished*^	Israel		Ashkenazi Jews*	68				11	89	6	94	67.7	32.4	66	34	41	59			66.9	33.1		
Kidd unpublished*^	Israel		Samaritans	36	21.8	78.2	0	57.7	42.3	30.3	69.7	51.3	48.8	94	6	32.5	67.5			60.3	39.7	34.7	65.3
Kidd unpublished*^	Israel		Sephardic Jews	25						12	88												
Kidd unpublished*^	Kenya		Masai	20				28	72														
Kidd unpublished*^	Laos		Lao Lhum	117				38	62	38.9	61.1	10	90	46	54	90.3	9.8			75	25		
Kidd unpublished*^	Mexico		Pima	51				83	17					84.9	15.1							20.6	79.4
Kidd unpublished*^	Nigeria		Hausa	36	14.9	74.3	10.8	36.8	63.2	27	73	10.5	89.5	35	65	94.9	5.1			24.3	75.7	33.3	66.7
Kidd unpublished*^	Nigeria		Ibo	44	17	73.4	9.6	37.5	62.5	11.1	88.9	16	84	26	74	99	1			17	83	42	58
Kidd unpublished*^	Pakistan		Pashtun	94						21.8	78.2												
Kidd unpublished*^	Russia		Chuvash	39				26.2	73.8	25	75	38.1	61.9	60	40	63.1	36.9			48.7	51.3		
Kidd unpublished*^	Russia		Khanty	44				26.1	73.9	25	75	24.5	75.5	49	51	78.6	21.4			87.2	12.8		
Kidd unpublished*^	Russia		Komi-Zyrian	44				12	88	6.8	93.2	53.2	46.8	46	54	53.2	46.8			74.4	25.6		
Kidd unpublished*^	Russia		Russians	31				26	74	24.2	75.8	47	53	69	31	54.6	45.5			70	30		
Kidd unpublished*^	South Korea		South Koreans	49				41	59	37.8	62.2	6	94	46.2	53.8	92.6	7.4			74.5	25.5		
Kidd unpublished*^	Tanzania		Chagga	41				42.2	57.8	19.8	80.2	18.3	81.7	37	63	95.6	4.4			26.7	73.3		
Kidd unpublished*^	Tanzania		Masai	19						13.2	86.8	15	85	25	75	97.5	2.5			29	71		
Kidd unpublished*^	Tanzania		Sandawe	39				28	72	10.3	89.7	28	72	20	80	92.5	7.5			21	79		
Kidd unpublished*^	Tanzania		Zaramo	35						9.7	90.3			24.3	75.7	97.4	2.6			21.8	78.2		
Kidd unpublished*^	US Afro		Afro-American	78	20.6	72.8	6.6	33.3	66.7	30.8	69.2	18.3	81.7	35	65	87.6	12.4			32.8	67.2	24	76
Kidd unpublished*^	US Amerindian		Pima	49	4.8	95.2	0													26.5	73.5		
Kim et al., 2000	South Korea		South Korean	128	14.05	81.25	3.9																
Kimura et al., 2002	Japan		Japanese	300	4.2	91.8	4																
Knott et al., 2010	Canada		Canadian	24				35	65														
Koehler et al., 2011	Germany		German	141				23.4	76.6									92.55	7.45				
Kõks et al., 2006	Estonia		Estonian	160				20	80	32	68	61	39			6	94	84	16	69	31	41	59
Kono et al., 1997	Japan		Japanese	93				37.15	62.95														
Kopeckova et al., 2008	Czech Republic		Czech	100	31	69	0	14.5	85.5											67	33		
Kordas et al., 2011	Mexico		Mexican children	220				48.2	51.8														
Kovanen et al., 2010	Finland		Finnish	511				19.15	80.85							52.55	47.45						
Kramer et al., 2007	Spain		Spanish	656	35.4	64.4	0.2															47.1	52.9
Kraschewski et al., 2009	Germany		German Caucasian	364				17.5	82.5							43.75	56.35	89.15	10.85				
Krebs et al., 1998	France		French Caucasian	52																64.45	35.45		
Krelling et al., 2008	Brazil		Brazilian females	62																45.9	54.1		
Kuhn et al., 1999	Germany		German male students	190																72.25	27.75		
Kukreti et al., 2006	South India	Bangalore	South Indian	145										63.1	36.9	55.15	44.85						
Kurt et al., 2011	Turkey		Turkish	60														77.5	22.5				
Laakso et al., 2005	Finland		Finnish	33				15.15	84.85							45.4	54.5	93.95	6.05				
Lafuente et al., 2008b	Spain		Spanish Caucasian	287				20.15	79.85	20.1	79.9							90.1	9.9	66.1	33.9		
Lahey et al., 2011	US mixed		US children 70% Euro	162	24.05	75.95	0																
Lai et al., 2010	China		Han Chinese	300																		42.85	57.15
Lakatos et al., 2002	Hungary		Hungarian Caucasian	95																		48.95	51.05
Lan et al., 2009	China		Han Chinese	112										43.3	56.7	87.5	12.5						
Lannfelt et al., 1992	Sweden		Swedish	53																71.75	28.25		
Lawford et al., 1999	Australia		Australian Caucasian	33				4.5	95.5														
Lawford et al., 2000	Australia		Australian Caucasian	50				18	82														
Lawford et al., 2005	Australia		Australian Caucasian	148												41.85	58.15						
Le Couteur et al., 1997	Australia		Australian Caucasian	200	27.75	71.25	0.5																
Lea et al., 2000	Australia		Australian Caucasian	182										72	28								

Study	Nation	Region	Ethnicity	N	9R	10R	other	A1	A2	B1	B2	D1	D2	C	T	C	T	in	del	ser	gly	C	T
Lee et al., 1997	South Korea		South Korean males	100				38	62														
Lee et al., 2003b	South Korea		South Korean	93				39.75	60.25														
Lee et al., 2003c	South Korea		South Korean females	101																		46	54
Lee et al., 2005	Taiwan		Taiwanese children	183				52.5	47.5					47.5	52.5								
Lee et al., 2007a	South Korea		South Korean females	264				40.5	59.5														
Lee et al., 2010a	US Afro		US mothers Afro	61	21.3	78.7	0																
Lee et al., 2010a	US Euro		US mothers Euro	136	27.2	72.8	0																
Lee et al., 2010b	Taiwan		Han Chinese	266				36.7	63.4														
Lee et al., 2011a	South Korea		South Korean	559				40.65	59.45											71.85	28.15		
Legro et al., 1995	US Hispanic		US Hispanic	180																52	48		
Leighton et al., 1997	Hong Kong		Hong Kong Chinese	230	7.65	88.05	2.2																
Leszczynska-Rodziewicz et al., 2005	Poland		Polish	366														89.3	10.7	71.25	28.75	46.9	53.1
Li & Kidd 2009*^	Greece		Greek	46						7.6	92.4												
Li & Kidd 2009*^	Israel		Palestinian	61						9.8	90.2												
Li & Kidd 2009*^	Italy		Italians	87						13.2	86.8												
Li & Kidd 2009*^	Kuwait		Kuwaiti	12						8.3	91.7			53.6	46.4	65.6	34.4			70	30		
Li & Kidd 2009*^	Sardinia		Sardinians	33						19.7	80.3			86.4	13.6	30.9	69.1			67.1	32.9		
Li & Kidd 2009*^	southern Africa		Lisongo	7						21.4	78.6			31.3	68.8	100	0			25	75		
Li et al., 1999	China		Han Chinese	196														92.5	7.5				
Li et al., 1999	UK		UK Caucasian	137				28.5	72.5									90	10				
Li et al., 2000	China		Han Chinese	304																		40.3	59.7
Li et al., 2002	China		Han Chinese	189				25.9	74.1									90.9	9.1	70.5	29.5		
Li et al., 2007*	Pakistan		Mohanna	49						15.3	84.7												
Li et al., 2007*	Pakistan		Negroid Makrani	25						16	84												
Li et al., 2007*	Pakistan		Hazara	97						27.8	72.2												
Li et al., 2007*	Somalia		Somali	16						12.5	87.5												
Li et al., 2008*	China		Baima Dee	15						63.3	36.7												
Li et al., 2008*	China		Hmong	18						19.4	80.6												
Li et al., 2008*	China		Kazakh	32						21.9	78.1												
Li et al., 2008*	China		Khamba	18						22.2	77.8												
Li et al., 2008*	China		Li	22						45.5	54.6												
Li et al., 2008*	China		Mongolian	58						33.6	66.4												
Li et al., 2008*	China		Qiang	22						20.5	79.6												
Li et al., 2008*	China		Uyghur	36						38.9	61.1												
Li et al., 2013	Germany		German Caucasian younger adults	479	25.4	74.6	0																
Li et al., 2013	Germany		German Caucasian older adults	809	25	75	0																
Lim et al., 2012	South Korea		South Korean males	114				39.95	60.05											71.9	28.1		
Limosin et al., 2002	France		French Caucasian	107				20.05	79.95														
Lin et al., 2001	Taiwan		Taiwanese females	133				34.2	65.8														
Linkowska et al., 2010	Poland		Polish males	51	17.6	82.4	0																
Liu et al., 2004	Taiwan		Taiwanese Chinese	72	8.35	86.15	2.8																
Lobo et al., 2010	Canada		Canadian Caucasian	218				21.15	78.85							53.35	46.65	89.35	10.65	69.05	30.95		
Lochman et al., 2012	Czech Republic		Czech males	213																72.95	26.95		
Lohoff et al., 2010	US Afro		Afro-American	257				34.45	65.55														
López-Castromán, 2009	Spain		Madrid Spanish	203				21.9	78.1														
López-Rodríguez et al., 2011	Spain		Spanish	119				24.35	75.65											64.5	35.5		
Lorenzo et al., 2007	Spain		Spanish	132																64	36		
Løvlie et al., 2001	South India	Chennai	Indian	22																52.25	47.75		
Lu et al., 1992	France		French Caucasian	214				2.9	97.1														
Lu et al., 1996	Taiwan		Ami	20				30	70	30	70												
Lu et al., 1996	Taiwan		Atayal	21				52.4	47.6	54.8	45.3												
Lu et al., 1996	Taiwan		Han Chinese	24				41.65	58.35	40	60												
Lu et al., 2006	China		Han Chinese	100																		39	61
Luijk et al., 2011	Netherlands		Dutch children	506				17.55	82.55														
Luijk et al., 2011	US mixed		US children	512				18.7	81.3														
Lung et al., 2006	China		Chinese	428																		35.3	64.7
Luo et al., 2005	US Hispanic		Mexican Americans	251				43.6	56.4	38.6	61.4							83.5	16.5				
Ma et al., 2008	China		Han Chinese	286																69.3	30.8		
Madrid et al., 2001	Honduras		Mayan males	301				27.95	72.05														
Magro et al., 2006	Portugal		Portugese	93				17	83														
Manki et al., 1996	Japan		Japanese	100	5	91	2													71	29		
Mant et al., 1994	UK Euro		UK Caucasian	97																62.9	37.1		
Marignac et al., 2006	Argentina		Argentinian Amerindian	47				42.1	57.8											50.05	50.05		

Study	Nation	Region	Ethnicity	N	9R	10R	other	A1	A2	B1	B2	D1	D2	C	T	C	T	in	del	ser	gly	C	T
Marignac et al., 2006	Argentina		Argentinian European	56				28.5	71.5											62.05	38.05		
Marignac et al., 2006	Argentina		Ayoreos	4				50	50											50	50		
Marignac et al., 2006	Argentina		Mapuches	21				42.85	57.15											62.05	37.95		
Marignac et al., 2006	Argentina		Mataco-Mataguayos	20				39.65	60.35											37.5	62.5		
Marignac et al., 2006	Argentina		Tehuelches	8				43.55	56.45											51.8	48.1		
Marignac et al., 2006	Chile		Pehuenche	6				25	75											83.35	16.65		
Marignac et al., 2006	Paraguay		Lenguas	5				40	60											71.45	28.55		
Markett et al., 2011a/b	Germany		German Caucasian	100				20.5	79.5							46	54						
Markett et al., 2013	Germany		German Caucasian	142												49	51.1						
Martineau et al., 1994	France		French Caucasian	50																72	28		
Martinez et al., 2001	US mixed		US no ethnicity data	31	20.95	79.05	0																
Marusin 2006*^	Russia		Russians	502	21.5	78.4	0.1																
Marusin 2006*^	Russia		Khanty	129	18.6	77.9	3.5																
Marusin 2006*^	Russia		Yakut	393	5.5	91.7	2.8																
Maude et al., 2001	UK		UK Caucasian	464														89	11				
Maziade et al., 1997	Canada		Canadian	79																70	30		
McAllister et al., 2005	US Euro		US Caucasian	27				40.1	59.9														
McAllister et al., 2008	US Euro		US Caucasian	21				43	57														
McGuire et al., 2011	US Afro		Afro-American	66				43	57									60.6	39.4	26.5	73.5		
McGuire et al., 2011	US Asian		Asian-American	226				36.6	63.4									86.4	13.6	74.7	25.3		
McGuire et al., 2011	US Euro		Euro-American	1279				19.4	80.6									90.7	9.3	67.4	32.6		
McGuire et al., 2011	US Hispanic		Hispanic-American	164				29.6	70.4									81.4	18.6	51.2	48.8		
Mileva-Seitz et al., 2012	Canada		Canadian Caucasian females	150				19.9	80.1							51.5	48.5	92	8				
Mill et al., 2006	New Zealand		NZ Euro children	1006	23	76	1																
Mill et al., 2006	UK		UK children	2145	25	74	1																
Mitchell et al., 2000	Australia		Australian Greeks	21	38.1	52.4	9.5																
Mitchell et al., 2000	Australia		Australian Yolngu	18	0	97.2	2.8																
Mitchell et al., 2000	Colombia		Colombian	27	0	100	0																
Mitchell et al., 2000	N Africa mixed		Mauritania/Algeria/Tunisia/Morocco	10	20	75	5																
Mitchell et al., 2000	Russia		Siberian Altai-Kizhi	48	5.2	86.5	8.3																
Mitchell et al., 2000	Russia		Siberian Evenki	61	3.3	78.7	18																
Mitchell et al., 2000	Russia		Siberian Ket	16	3.1	93.8	3.1																
Mitchell et al., 2000	Russia		Siberian Sel'kup	29	20.7	70.7	8.6																
Mitchell et al., 2000	Russia		Russian Chuvash	33	10.6	87.9	1.5																
Mitchell et al., 2000	US Native American		US Native American	19	24	76	0																
Mitsuyasu et al., 1999	Japan		Japanese	80																		41.05	58.95
Mitsuyasu et al., 2001	Japan		Japanese	210																		38.1	61.9
Miyake et al., 1999	Japan		Japanese children	104				26.45	73.55														
Mochi et al., 2003	Italy		Italian	97	28.4	69.6	2																
Monakhov et al., 2008	Russia		Russian	364				18.65	81.35					69.6	30.4	46.55	53.45						
Montag et al., 2010a	Germany		German Caucasian	768				19.65	80.35														
Montag et al., 2010b	Germany		German Caucasian	161				19.55	80.45														
Montag et al., 2012	Germany		German Caucasian	105												45.75	54.25						
Morell et al., 1993	Germany		German	100																70	30		
Nacak et al., 2012	Turkey		Turkish	130				16.55	83.45	16.55	83.45												
Najafabadi et al., 2005	Iran		Iranian	130				9.2	90.8														
Nakagawa et al., 2008	Japan		Japanese	1069				36.6	63.4														
Nakajima et al., 2007	Japan		Japanese	569																		59	41
Nakatome et al., 1995	Japan		Japanese	176	6.3	91.2	2.5																
Nanko et al., 1993a/b	Japan		Japanese	81																73	27		
Neiswanger et al., 1995	US Euro		US Caucasian	30				7	93														
Nelis et al., 2009*	Estonia		Estonians	975				19.6	80.4											70.9	29.1		
Nemoda et al., 2010	US mixed		US young adults 67% Euro	97	26.5	69.4	4.1	25.3	74.8	14.4	85.6	49.5	50.5									39.35	60.55
Nicolini et al., 1996	Mexico		Mexican	54				52.5	47.5											60	40		
Nimgaonkar et al., 1993	US Euro		US Caucasian	61																62	38		
Nimgaonkar et al., 1996	US Afro		US Afro-American neonates	60																34	66		
Nimgaonkar et al., 1996	US Afro		US Afro-American adults	63																29	71		
Nimgaonkar et al., 1996	US Euro		US Caucasian neonates	100																68	32		
Nimgaonkar et al., 1996	US Euro		US Caucasian adults	65																65	35		
Nisoli et al., 2007	Italy		Italian	54				14.1	75.9														
Noble & Paredes, 1993	US Euro		US Caucasian	69				30.4	69.6														
Noble et al., 1998a	US Euro		Caucasian	45				7.8	92.2														
Noble et al., 1998b	US Euro		US Caucasian boys	119				19.75	80.25	15.95	84.05												

Study	Nation	Region	Ethnicity	N	9R	10R	other	A1	A2	B1	B2	D1	D2	C	T	C	T	in	del	ser	gly	C	T
Noble et al., 2000	US Euro		US Caucasian	85				11.15	88.85	9.45	90.55							90	10				
Nothen et al., 1992	Germany		German	69				15	85														
Nunokawa et al., 2010	Japan		Japanese	595																72	28		
O'Hara et al., 1993	US Afro		US Black	22				40.9	59.1	31.8	68.2												
O'Hara et al., 1993	US Euro		US White	106				16	84	10.4	89.6												
Ohara et al., 1996	Japan		Japanese	121																51.25	48.75		
Okuyama et al., 1999	Japan		Japanese	269																		40.5	59.5
Oliveri et al., 2000	Italy		Italian	134				14.65	85.45	10.65	89.35							95.5	4.5				
Oruc et al., 1996 / 1997	Croatia		Croatian	32																68.8	31.2		
Ovchinnikov et al., 1999	Russia		Russian Slavic surnamed males	76				20.4	79.6	13.85	86.15												
Paclt et al., 2010	Czech Republic		Czech boys	317				14.7	85.3														
Pakhomova et al., 2011	Russia		Russian	103				16.1	84.9														
Palmatier et al., 1999*^	Ethiopia		Ethiopian Jews*	32														75	25				
Palmatier et al., 1999*^	Mexico		Pima	50						83.7	16.3	2	98										
Parsian & Zhang 1997a	US Euro		US Caucasian	87	26.4	73	0.6																
Parsian et al., 1991	US Euro		US Caucasian	39								61	39										
Parsian et al., 1995 / 1997b / 2000	US Euro		US Caucasian	88														91	9	26.4	1.65		
Parsons et al., 2007	Spain		Northern Spanish	153				23.5	76.5									94	6				
Pastorelli et al., 2001	Italy		Italian	64				13.25	86.75	8.65	91.45												
Pelka-Wysiecka et al., 2012	Poland		Polish Caucasian	406	22.9	77	0																
Perkins et al., 2008	US Euro		US European	99												44.4	55.5						
Persico et al., 1993	US mixed		US no ethnicity data	48	68.8	25	0																
Persico et al., 1996	US Euro		US Caucasians	101				11.6	88.4	11.6	88.4	55.95	44.05										
Persico et al., 1998	Italy		Italian	54	39.8	59.2	1																
Pohjalainen et al., 1998/1999	Finland		Finnish	52				15.75	84.25									93.3	6.7				
Potvin et al., 2009	Canada		Canadian	36																59.65	40.25		
Prasad et al., 2010	North India	New Delhi	Indian males	60				22	78	26.5	73.5							65	35				
Reddy et al., 2007	South India	Andhra Pradesh	Akuthota	31				37.1	62.9														
Reddy et al., 2007	South India	Andhra Pradesh	Balija	34				34.4	65.6														
Reddy et al., 2007	South India	Andhra Pradesh	Brahmin	21				28.6	71.4														
Reddy et al., 2007	South India	Andhra Pradesh	Chakali	29				50	50														
Reddy et al., 2007	South India	Andhra Pradesh	Devanga	62				33.1	66.9														
Reddy et al., 2007	South India	Andhra Pradesh	Dudekula Muslim Indian	26				40.4	59.6														
Reddy et al., 2007	South India	Andhra Pradesh	Ediga	32				27.6	72.4														
Reddy et al., 2007	South India	Andhra Pradesh	Ekila	35				34.3	65.7														
Reddy et al., 2007	South India	Andhra Pradesh	Erukala Proto-Australoid Indian	80				26.3	73.7														
Reddy et al., 2007	South India	Andhra Pradesh	Gandla	21				26.9	73.1														
Reddy et al., 2007	South India	Andhra Pradesh	Jangam	14				32.1	67.9														
Reddy et al., 2007	South India	Andhra Pradesh	Kamma	48				33.3	66.7														
Reddy et al., 2007	South India	Andhra Pradesh	Kapu	24				22.9	77.1														
Reddy et al., 2007	South India	Andhra Pradesh	Kshatriya	25				28	72														
Reddy et al., 2007	South India	Andhra Pradesh	Kurava	50				29	71														
Reddy et al., 2007	South India	Andhra Pradesh	Madiga	65				39.2	60.8														
Reddy et al., 2007	South India	Andhra Pradesh	Mala	50				38	62														
Reddy et al., 2007	South India	Andhra Pradesh	Mangali	13				42.3	57.7														
Reddy et al., 2007	South India	Andhra Pradesh	Panta	47				26.3	73.7														
Reddy et al., 2007	South India	Andhra Pradesh	Pokanati	39				33.3	66.7														
Reddy et al., 2007	South India	Andhra Pradesh	Sheik Muslim Indian	21				38.1	61.9														
Reddy et al., 2007	South India	Andhra Pradesh	Sugali Caucasoid Indian	39				30.8	69.2														
Reddy et al., 2007	South India	Andhra Pradesh	Thogata	16				34.4	65.6														
Reddy et al., 2007	South India	Andhra Pradesh	Vadde	40				33.8	66.2														
Reddy et al., 2007	South India	Andhra Pradesh	Vanne	32				31.7	68.3														
Reddy et al., 2007	South India	Andhra Pradesh	Vysya	19				28.9	71.1														
Reddy et al., 2007	South India	Andhra Pradesh	Yadava	31				41.9	58.1														
Reddy et al., 2007	South India	Andhra Pradesh	Yanadi Proto-Australoid Indian	106				22.6	77.4														
Reimers et al., 2012	Ireland		Irish	519																64.1	35.9		
Reuter et al., 2006	Germany		German Caucasian	92				19.5	80.4														
Rietschel et al., 1993	Germany		German	100																70	30		
Rodriguez-Jimenez et al., 2006	Spain		Spanish	83												40.4	59.6						
Ronai et al., 2001b	Hungary		Hungarian Caucasian	119																		43.5	56.5
Rosenberg et al., 2002*	Algeria		Mozabite	30										52	48					48	52		
Rosenberg et al., 2002*	Brazil		Karitiana	54										61	39								
Rosenberg et al., 2002*	Central African Republic		Mandenka	24										33	67					32.8	67.2		

Study	Nation	Region	Ethnicity	N	9R	10R	other	A1	A2	B1	B2	D1	D2	C	T	C	T	in	del	ser	gly	C	T
Rosenberg et al., 2002*	China		Dai	10										50	50					70	30		
Rosenberg et al., 2002*	China		Daur	9										50	50					72	28		
Rosenberg et al., 2002*	China		Hezhe	9										44	56					83	17		
Rosenberg et al., 2002*	China		Lahu	10										50	50								
Rosenberg et al., 2002*	China		Miao	10										35	65					75	25		
Rosenberg et al., 2002*	China		Mongolian	10										60	40					85	15		
Rosenberg et al., 2002*	China		Naxi	9										39	61					78	22		
Rosenberg et al., 2002*	China		Oroqen	10										40	60					80	20		
Rosenberg et al., 2002*	China		She	10										60	40					75	25		
Rosenberg et al., 2002*	China		Tu	10										25	75					80	20		
Rosenberg et al., 2002*	China		Tujia	10										65	35					75	25		
Rosenberg et al., 2002*	China		Uyghur	10										50	50					65	35		
Rosenberg et al., 2002*	China		Xibe	9										50	50					78	22		
Rosenberg et al., 2002*	China		Yi	10										25	75					90	10		
Rosenberg et al., 2002*	Colombia		Amerindians	13										19	81								
Rosenberg et al., 2002*	France		Basque	24										69	31					65	35		
Rosenberg et al., 2002*	France		French	29										76	24					64	36		
Rosenberg et al., 2002*	Israel		Palestinian	51										67	33								
Rosenberg et al., 2002*	Italy		Italian	8										81	19					75	25		
Rosenberg et al., 2002*	Pakistan		Balochi	25										54	46					62	38		
Rosenberg et al., 2002*	Pakistan		Balochi	25										44	56					74	26		
Rosenberg et al., 2002*	Pakistan		Brahui	25										62	38					60	40		
Rosenberg et al., 2002*	Pakistan		Burusho	25										60	40					60	40		
Rosenberg et al., 2002*	Pakistan		Hazara	24										52	48					60	40		
Rosenberg et al., 2002*	Pakistan		Kalash	25										40	60					38	62		
Rosenberg et al., 2002*	Pakistan		Sindhi	25										58	42					74	26		
Rosenberg et al., 2002*	Papua New Guinea		Papua New Guinean	17										18	82					53	47		
Rosenberg et al., 2002*	Sardinia		Sardinians	28										75	25					70	30		
Rosenberg et al., 2002*	UK		Orkney Islanders	16										84	16					66	34		
Rosenberg et al., 2002*^	Palestine		Palestinian	51																63	37		
Rosenberg et al., 2005*	Kenya		Bantu	12										25	75					17	83		
Rosenberg et al., 2005*	Namibia		San	6										25	75					17	83		
Rosenberg et al., 2005*	southern Africa		Bantu	8										25	75					19	81		
Rosenberg et al., 2005*/2006	Central African Republic		Biaka	31										16	84					44	56		
Rosenberg et al., 2006*	Israel		Bedouin	48										54	46					61	39		
Rosenberg et al., 2006*	Italy		Italian	13										62	38					62	38		
Rosenberg et al., 2006*	Japan		Japanese	27										46	54								
Rosenberg et al., 2006*	Pakistan		Pashtun	23										67	33								
Rosenberg et al., 2006*^	China		Lahu	10																85	15		
Rosenberg et al., 2006*^	Japan		US Japanese*	28																71	29		
Rosmond et al., 2001	Sweden		Swedish males	263										70.1	29.9								
Roussos et al., 2008	Greece		Greek Caucasian	101																63.9	36.1		
Rowe et al., 2001	US Euro		US Euro-American	93	0	68.8	31.2																
Roy et al., 2011	South India	Chennai	Chennai Indians	717				30.4	69.6														
Rybakowski et al., 2001	Poland		Polish	94																75.5	24.5		
Safarinejad et al., 2010	Iran		Iranian males	266	5.85	86.25	4																
Saha et al., 1994	China		Chinese males	125																70	30		
Sailors et al., 2010	US Afro		US Afro-American	440				37	62			21.5	78.5										
Sailors et al., 2010	US Euro		US Caucasian	456				23	77			53.5	46.5										
Sailors et al., 2010	US Hispanic		US Hispanic	359				38.5	61.5			36.5	63.5										
Sáiz et al., 2010	Spain		Spanish Caucasian	395	32.95	63.75	2											87	13	65.3	34.7		
Sakai et al., 2007	US Euro		US Caucasian	84				16.7	83.4														
Sakai et al., 2007	US Hispanic		US Hispanic	50				40	60														
Samochowiec et al., 2000	Germany		German	192				15.9	84.1									90.5	9.5				
Samochowiec et al., 2008	Poland		Polish Caucasian	150	24	76	0	18	82									91	9				
Sander et al., 1997	Germany		German	197				16.9	83.2									90.6	9.4				
Sanders et al., 1993	US Euro		US Caucasian	51				17.65	82.35														
Sanders et al., 2008	US Euro		US Caucasian	1908										70.5	29.5	46.75	53.25	90.5	9.5				
Sano et al., 1993	Japan		Japanese	107	4.2	93	2.8																
Santovito et al., 2008	Italy		Italian	204	34.05	60.05	4																
Santovito et al., 2008	Ivory Coast		Ivory Coast	73	14.35	71.85	11.7																
Saraswathy et al., 2009a	North India	Manipur	Kom	38				11	89	15	85	85	15										
Saraswathy et al., 2009a	North India	Manipur	Meitei	46				28.2	71.6	36.6	63.2	6.6	93.2										

Study	Nation	Region	Ethnicity	N	9R	10R	other	A1	A2	B1	B2	D1	D2	C	T	C	T	in	del	ser	gly	C	T
Saraswathy et al., 2009a	North India	Manipur	Paite	44				51.6	48.2	46.5	53.3	86.1	13.7										
Saraswathy et al., 2009a	North India	Manipur	Thadou	45				10.6	89.4	45.4	54.6	86.4	13.6										
Saraswathy et al., 2009b	South India	Andhra Pradesh	Brahmin	50						27.9	72.1	34.9	65.1										
Saraswathy et al., 2009b	South India	Andhra Pradesh	Kolam	53						44.3	55.7	19.3	80.7										
Saraswathy et al., 2009b	South India	Andhra Pradesh	Nayakpod	49						41.4	58.7	19.6	80.5										
Saraswathy et al., 2009b	South India	Andhra Pradesh	Thoti	52						35.4	64.6	23	77										
Sato et al., 2009	Japan		Japanese	224																74.6	25.4		
Savoye et al., 1998	France		NW French	86																66.25	33.75		
Savoye et al., 1998	France		NW French	163				18.45	81.65														
Savoye et al., 1998	France		NW French	205				17.05	82.95														
Scherk et al., 2009	Germany		German	16	31.25	68.75	0																
Schosser et al., 2010	Austria		Austrian Caucasian	89																65.5	34.5		
Segman et al., 1999	Israel		Jewish 51% Ashkenazi*	117																72.7	27.3		
Segman et al., 2002	Israel		Jewish 40% Ashkenazi*	95	30.55	69.45	0																
Sery et al., 2001	Czech Republic		Czech Caucasians	132				23.1	76.9														
Sery et al., 2006	Czech Republic		Czech Caucasians	153				15.05	84.95														
Shaikh et al., 1996	UK Euro		UK Caucasian	109																56	44		
Shepherd et al., 2002	Australia		Australian	275																70	30		
Shinohara et al., 2004	Japan		Japanese females	115	3.9	93.9	2.2																
Shook et al., 2011	US mixed		US children 46% Caucasian	26	19.25	80.75	0																
Shriver et al., 2005*^	DR Congo		Mbuti	37						0	100												
Shriver et al., 2005*^	South India	Andhra Pradesh	Brahmin	11						27.3	72.7												
Shriver et al., 2005*^	South India	Andhra Pradesh	Mala	11						18.2	81.8												
Shriver et al., 2005*^	Mexico		Nahuas	20						65	35												
Shriver et al., 2005*^	Peru		Quecha	20						45	55												
Shriver et al., 2005*^	Russia		Altaian	18						33.3	66.7												
Shriver et al., 2005*^	Sierra Leone		Mende	22						9.1	90.9												
Shriver et al., 2005*^	Spain		Spaniards	19						10.5	89.5												
Shriver et al., 2005*^	Tanzania		Borunge	19						21.1	79												
Shriver et al., 2005*^	US Afro		Afro-Americans	42						13.1	86.9												
Shriver et al., 2005*^	US Euro		US Europeans	41						12.2	87.8												
Shriver et al., 2005*^	US Hispanic		Puerto Rican	20						7.5	92.5												
Sieminska et al., 2009	Poland		Polish Caucasian	158	22.45	76.85	0.3	22.5	77.5														
Simsek et al., 2005a	Oman		Omani	110	31.35	58.65	5.9																
Singleton et al., 1998	UK		UK Caucasian	117				24	76														
Sipila et al., 2010*	Finland		Finnish	653				19.6	80.4					65.8	34.2								
Sivagnanasundaram et al., 2000	UK Euro		UK Caucasian	56																65.3	34.7		
Soma et al., 2002	Japan		Japanese	181																67.35	32.55		
Souery et al., 1996	Belgium		Belgian	48	24.95	75.05	0													72.5	27.5		
Spangler et al., 2009	Germany		German Caucasian infants	106																		49.5	50.5
Squassina et al., 2011	Sardinia		Sardinian	277				14.8	85.2														
Staddon et al., 2005	Spain		Basque	156																67.5	32.5		
Stefan et al., 2009	Germany		German	98				18	82							50	50						
Stevenson et al., 2010	UK		UK 3yos	104	60.6	39.4	0																
Stevenson et al., 2010	UK		UK 8-9yos	107	70.1	29.9	0																
Stice et al., 2012	US mixed		US 78% Euro 16% mixed race	155	23.75	76.25	0	20.9	79.1									87.1	12.9				
Stober et al., 1998	Germany		German Caucasian	290														90.5	9.5				
Strobel et al., 2002	Germany		German	276																		49.8	50.2
Suarez et al., 1994	US Euro		US Caucasian	87				15.35	84.65	13.2	86.8												
Suda et al., 2009	Japan		Japanese	123				48	52									76	24				
Swagell et al., 2012	Australia		Australian Caucasian	227				16.95	83.05							42.5	57.5						
Sysoeva et al., 2010	Russia		Russian Caucasian males	43	26.15	73.85	0	26.75	73.25														
Szantai et al., 2005*	Hungary		Hungarian	598																		46.5	53.5
Szczepankiewicz et al., 2007	Poland		Polish	350														89.1	10.9	71.4	28.6	47.55	52.45
Szekely et al., 2011	Hungary		Hungarian Caucasian	221	32.55	67.55	0	17.45	82.45													45.5	54.5
Szilagyi et al., 2005	Hungary		Hungarian Caucasian	362																		45.7	54.3
Takahashi et al., 2008	Japan		Japanese	29																69	31.1		
Talkowski et al., 2008a	US Euro		US Caucasian neonates	500																62.7	37.3		
Tallerico et al., 1999	US Euro		US Caucasian	51														91	9				
Tan et al., 2003	Singapore		Singaporean	216				39.35	60.65	41.9	58.1												
Tanaka et al., 1995/1996	Japan		Japanese	70																74.05	26.05		
Tee et al., 2011a	Malaysia		Malaysian	157																66.85	33.15		
Thomas et al., 2000	Hong Kong		Hong Kong Han Chinese	174				51.1	48.9														

Study	Nation	Region	Ethnicity	N	9R	10R	other	A1	A2	B1	B2	D1	D2	C	T	C	T	in	del	ser	gly	C	T
Thomas et al., 2001	Hong Kong		Hong Kong Han Chinese	519				44.95	55.05														
Thompson et al., 1997	UK		UK Caucasian	44				22.75	77.25														
Tishkoff et al., 1998*	Denmark		Danish	43				14	86	11.6	88.4	58.8	41.2							60.8	39.2		
Tishkoff et al., 1998*	Ethiopia		Ethiopian Jews*	21						8.6	91.4	38.1	61.9										
Tishkoff et al., 1998*	South Korea		South Koreans	127				40.9	59.1			6.3	93.7										
Treister et al., 2011	Israel		Jewish 72% Ashkenazi*	191	41.2	55.7	3.1																
Tsai et al., 2002a	China		Chinese females	112				35.3	64.8														
Tsai et al., 2002b	Taiwan		Han Chinese	112				42	58														
Tsuchimine et al., 2012	Japan		Japanese	1067				34.9	65.1									83.5	16.5				
Tsutsumi et al., 2011	Japan		Japanese	384												93.85	6.05						
Turner et al., 1997	UK		UK Caucasian	307				19.5	80.5														
Ujike et al., 2003	Japan		Japanese	160	7.55	92.55	0																
Ujike et al., 2009	Japan		Japanese	243				38.75	61.25									86.7	13.3	71.4	28.5	45.25	54.65
Valente et al., 2011	Brazil		Brazilian	297	26.4	73.6	0																
van de Giessen et al., 2009	Netherlands		Dutch	77	24.05	75.95	0																
van der Zwaluw et al., 2011 / 2012	Netherlands		Dutch adolescents	252				18	82														
van Munster et al., 2010a	Netherlands		Dutch 65+	409	24.5	75.5	0	18.5	81.5							48.5	52.5	90	10	65.5	34.5		
Vandenbergh et al., 2002a	US Afro		US Afro-American	21	21.45	78.55	0																
Vandenbergh et al., 2002a	US Euro		US Euro-American	291	24.55	75.45	0																
Vaske et al., 2009a	US Afro		US Afro-American	402	17.5	82.5	0																
Vaske et al., 2009a	US Euro		US Euro-American	1552	24.15	75.85	0																
Ventriglia et al., 2002	Italy		Italian Caucasian	138																67.1	33		
Verde et al., 2011	Spain		Spanish	80				21.95	78.05														
Verga et al., 1997	Japan		Japanese	97																			
Vijayan et al., 2007	South India	Kerala	South Indian	193				35	65	26	74	40.5	59.5	49	51								
Virgos et al., 2001	Spain		Spanish	278																70.9	29.1		
Vishwanathan et al., 2004*	South India	Tamil Nadu	Badaga	51				41	59	34.3	65.7	34.8	65.2										
Vitale et al., 2008	Italy		Italian	158																55	45		
Voisey et al., 2012a	Australia		Australian Caucasian	250				16.95	83.05					70.1	29.9	42.5	57.5	88.7	11.3				
Wacker et al., 2005	Germany		German Caucasian	504				20.45	79.55														
Walter et al., 2011a	Germany		German Caucasian	664				20.5	79.5														
Wang et al., 2007	China		Han Chinese	66	5.3	85.6	4.5																
Wang et al., 2012a	China		Han Chinese	312				37.65	62.35														
Wiebe et al., 2009	US Euro		US Caucasian babies	98				20.95	79.05														
Wiebe et al., 2009	US Euro		US Caucasian preschoolers	58				25.05	74.95														
Wiener et al., 2011	US mixed		US	65				21.85	78.15														
Wu et al., 2000	US Afro		Afro-Americans	94				36.95	63.05	15.85	84.15												
Wu et al., 2000	US Hispanic		Mexican Americans	97				42.05	57.95	38.15	61.85												
Xing et al., 2003	China		Han Chinese	206																		39.05	60.95
Xu et al., 2007	US Euro		US Caucasian	188												52.95	47.05						
Yamada et al., 2003	Japan		Japanese	2228																		40.6	59.4
Yang et al., 1993	China		Han Chinese	98																71.4	28.6		
Yang et al., 2007	US Euro		US Caucasian	414				20.4	79.6	15.2	84.8	79.6	20.4			53.7	46.3	89.2	10.8				
Yang et al., 2008	South Korea		South Korean boys	95																		51.05	48.95
Yeh et al., 2010	Taiwan		Taiwanese females	558																66.55	33.45		
Yeh et al., 2012	Taiwan		Taiwanese students	134																66.4	33.6		
Yoshida et al., 2001	Japan		Japanese	198				42.5	57.5									85	15				
Yoshimura et al., 2003	Japan		Japanese males	212										59.9	40.1	94.4	5.6			73.1	26.9	37	63
Young et al., 2002	Australia		Australian Caucasian	51				6.9	93.1														
Zai et al., 2010	Canada		Canadian Caucasian	167																63.2	36.8		
Zai et al., 2012	Canada		Canadian 78% Caucasian	128	26	73	1	17.5	82.5			54	46			54	46	83	17				
Zappia et al., 2001	Italy		Italian	202				14.6	85.4														
Zhang et al., 2011	China		Han Chinese	768																71.3	28.7		
Zhu et al., 2012	China		Chinese students	478												92.6	7.4						
